# Numerical Assessment of the Stamping Formability of SS304 Metallic Bipolar Plates with Side-Boss Parallel Flow Channels

**DOI:** 10.3390/ma19163362

**Published:** 2026-08-07

**Authors:** Zhonglan Hou, Yibo Zhang, Pengyan Guo, Mohammad Hossein Yazdi, Perk Lin Chong, Fuwang Zhou, Haining Bu, Xiao Zhu, Jie Shen, Zhen Zhang, Yuguo Gao

**Affiliations:** 1 School of Mechanical Engineering, North China University of Water Resources and Electric Power, Zhengzhou 450045, China; 17335214336@163.com (Y.Z.); 19146846476@163.com (F.Z.); 16634864155@163.com (H.B.); 15518571513@163.com (X.Z.); shenjie@ncwu.edu.cn (J.S.); zhangzhen@ncwu.edu.cn (Z.Z.); gaoyuguo@ncwu.edu.cn (Y.G.); 2 College of Engineering and Technology, University of Technology and Applied Sciences, Al-Khuwair, P.O. Box 74, Muscat 133, Oman; mohammad.hossein@utas.edu.om; 3 School of Computing, Engineering & Digital Technologies, Department of Engineering, Teesside University, Middlesbrough TS1 3BX, UK; p.chong@tees.ac.uk

**Keywords:** proton exchange membrane fuel cell, metallic bipolar plate, stamping process, side-boss, finite element simulation

## Abstract

Side-boss parallel channels can enhance reactant transport and water removal in proton exchange membrane fuel cells, but their local protrusions increase the forming difficulty of ultra-thin metallic bipolar plates. This study numerically investigates the single-step stamping formability of 0.1 mm SS304 bipolar plates using Dynaform. The effects of side-boss number, side-boss height, and punch speed were evaluated through thickness distribution, maximum thinning ratio, and forming-limit-diagram states. The results show that side-boss height is the dominant geometric factor. Increasing the height from 0 to 0.75 mm raises the maximum thinning ratio from 17.69% to 29.87% and shifts the critical deformation regions from the conventional channel bottom toward the side-boss roots, boss tops, transition fillets, and channel corners. The number of side-boss sets produces a non-monotonic thinning response, with maximum thinning ratios ranging from 19.54% to 27.76% for the modified configurations. Punch speeds of 200, 500, and 800 mm/s yield comparable thinning levels of 24.60%, 23.09%, and 23.16%, respectively, whereas the value increases to 26.30% at 1100 mm/s. Under the present forming conditions, these findings establish a quantitative relationship between side-boss geometry, material-flow restriction, and strain localization, and provide practical guidance for geometry selection and stamping-process design of metallic bipolar plates.

## 1. Introduction

Proton exchange membrane fuel cells (PEMFCs) are regarded as one of the most promising electrochemical energy-conversion technologies for low-carbon transportation because of their high power density, high energy-conversion efficiency, rapid start-up capability, relatively low operating temperature, and near-zero local emissions [1,2]. Among the various components of a PEMFC stack, bipolar plates play a particularly critical role because they simultaneously provide mechanical support, collect and conduct current, distribute reactant gases, remove generated water, and assist thermal management. Their structural design and manufacturing quality therefore have direct effects on cell performance, stack reliability, and production cost. In addition, bipolar plates account for a considerable proportion of the stack cost, weight, and volume, making their lightweight and high-precision fabrication a key issue for PEMFC commercialization [2,3].

Metallic bipolar plates, especially stainless-steel bipolar plates, have attracted extensive attention as alternatives to conventional graphite plates owing to their high electrical and thermal conductivity, good gas impermeability, excellent mechanical strength, relatively low material cost, and suitability for thin-sheet mass production [4]. Stainless-steel bipolar plates with thicknesses of approximately 0.1 mm or even lower can be produced by plastic-forming processes, which is advantageous for reducing stack volume and improving power density. However, the micro-channel structures required for gas distribution and water removal introduce severe local deformation during forming. Excessive thinning, wrinkling, springback, insufficient channel filling, and fracture may occur when ultra-thin sheets are subjected to complex micro-scale geometries. Therefore, the forming quality of metallic bipolar plates is not only a manufacturing concern but also a prerequisite for ensuring stable electrochemical performance and long-term stack durability.

Various forming technologies have been developed to improve the manufacturability of metallic bipolar plates. Bong et al. [5] proposed a two-stage forming method for ferritic stainless-steel plates, while Xu et al. and Zhong et al. demonstrated that multi-stage deformation can increase the achievable microchannel geometry and reduce local forming severity in titanium sheets [6,7]. Hydroforming, rubber-pad forming, micro-roll forming, and hot stamping have also been investigated as alternatives to conventional rigid-die stamping [7,8,9,10,11,12,13,14,15]. These processes redistribute the required deformation through different pressure paths, contact conditions, temperatures, or intermediate shapes. Nevertheless, stamping remains attractive for high-volume production because of its short cycle time, mature tooling route, and compatibility with ultra-thin metal sheets.

Recent studies have progressively expanded forming assessment beyond a single thinning value. Seddighi et al. experimentally and numerically investigated wrinkling control in a 0.1 mm SS304 parallel-serpentine plate using rubber blank-holder restraint [16]. Guo et al. demonstrated the importance of forming temperature and lubrication for the dimensional accuracy of ultra-thin stainless-steel bipolar plates [10]. Zhang et al. examined failure initiation in micro-stamped 316L sheets using measured tool geometry and an experimental forming-limit diagram [17]. More recently, Acar et al. combined physical stamping and three-dimensional simulation for SS316L and CP-Ti plates and evaluated fracture, wrinkling, thinning, channel depth, blank-holder restraint, press velocity, and springback [18]. These investigations show that bipolar-plate quality must be assessed through coupled defect, thickness, and dimensional criteria. However, the studied geometries remain conventional parallel, serpentine, or parallel-serpentine channels rather than repeated local sidewall bosses.

Numerical process design has also become more systematic and computationally efficient. Yang et al. incorporated material and tooling variability into robust process design, while Khatir et al. and Modanloo et al. applied design-of-experiments and response-surface methods to geometric and process optimization [19,20,21]. Gassler et al. recently introduced a GPU-accelerated one-step solver for metallic bipolar-plate stamping and obtained qualitatively consistent strain and thinning distributions at substantially lower computational cost, although small quantitative differences from incremental simulations remained [22]. Lan et al. further optimized forming parameters and forming strategy for an ultra-thin super-ferritic stainless-steel bipolar plate [23]. These advances improve screening efficiency, but they continue to focus primarily on conventional channel profiles, global process variables, or numerical acceleration. The additional bending–stretching and material-flow restriction introduced by repeated sidewall protrusions has not been systematically isolated.

Experimental and numerical studies have established that channel depth, channel-to-rib ratio, fillet radius, die clearance, friction, blank-holder force, press force, and forming sequence strongly influence thickness distribution, channel filling, wrinkling, springback, and fracture [16,18,19,20,21,23,24,25,26,27,28,29,30,31,32,33,34,35]. Severe deformation is frequently concentrated at high-curvature sheet–tool contact transitions and in regions where material draw-in is restricted. However, the geometric variables in these studies generally modify the main channel cross-section or the overall forming route rather than introducing discrete protrusions repeatedly along a channel sidewall. In parallel, performance-oriented PEMFC research has demonstrated the fluid–dynamic benefits of local disturbances. Partially blocked channels, streamlined baffles, variable-height blocks, three-dimensional obstacles, tapered-baffle arrangements, and auxiliary channels can enhance transverse convection, under-rib oxygen transport, water removal, and current-density uniformity [36,37,38,39,40,41,42,43,44]. Li et al. optimized a blocked regulated tri-serpentine field with variable block heights and auxiliary channels [42], while Nguyen and Kim developed a staggered trap/block structure within a serpentine field [44]. These studies demonstrate the electrochemical value of local obstacles, but their evaluation criteria are mainly pressure drop, oxygen transport, liquid-water distribution, current density, and power output rather than the stampability of ultra-thin metallic sheets.

The local features in most blocked or baffled flow fields are also geometrically different from the present design. Many occupy the channel centre or a substantial portion of the cross-section, are coupled with globally serpentine or tapered paths, or modify the overall channel trajectory. By contrast, each side-boss investigated here is a discrete transverse protrusion extending from the sidewall of an otherwise straight parallel channel. The global channel centerline, principal flow direction, main channel count, and nominal channel depth are retained, while additional boss root, boss top, and transition regions are introduced. Consequently, two mature research directions remain insufficiently connected. Metallic bipolar-plate forming studies have established deformation rules for conventional channels and global process variables. Flow-field-performance studies have established that local obstacles can improve mass transfer and water management. What remains insufficiently quantified is the additional single-step stamping demand generated by repeated performance-motivated sidewall protrusions in a 0.1 mm SS304 parallel-channel plate.

This study addresses that manufacturing gap through a Dynaform-based comparative assessment. The contributions are fourfold. First, a distinct side-boss geometry is defined in which discrete sidewall protrusions are introduced while the global parallel path is preserved. Second, the separate effects of side-boss number, side-boss height, and punch speed are quantified under common contact and blank-holder conditions. Third, the migration of minimum-thickness and high-risk regions from the conventional channel bottom toward the boss root, boss top, transition fillet, and channel corner is identified using thickness distribution, maximum thinning ratio, and estimated-FLD states. Fourth, the quantified trends are converted into manufacturing-oriented guidance for geometry selection and process design. The novelty of this work lies in isolating and quantifying the single-step stamping penalty introduced by repeated local sidewall bosses while retaining the global parallel-channel path, a manufacturability issue not systematically addressed in previous conventional channel-forming or performance-oriented obstacle-flow-field studies.

## 2. Numerical Model and Formability Evaluation

The numerical framework combines geometric parameterization, an SS304 elastic-plastic material model, contact and loading conditions, forming-limit-based assessment, mesh convergence, and comparison with published stamping evidence. This unified framework is used to quantify thickness evolution, local-necking tendency, and critical-region migration under controlled changes in side-boss geometry and punch speed.

### 2.1. Geometric Model and Parameter Design

**Figure 1:**
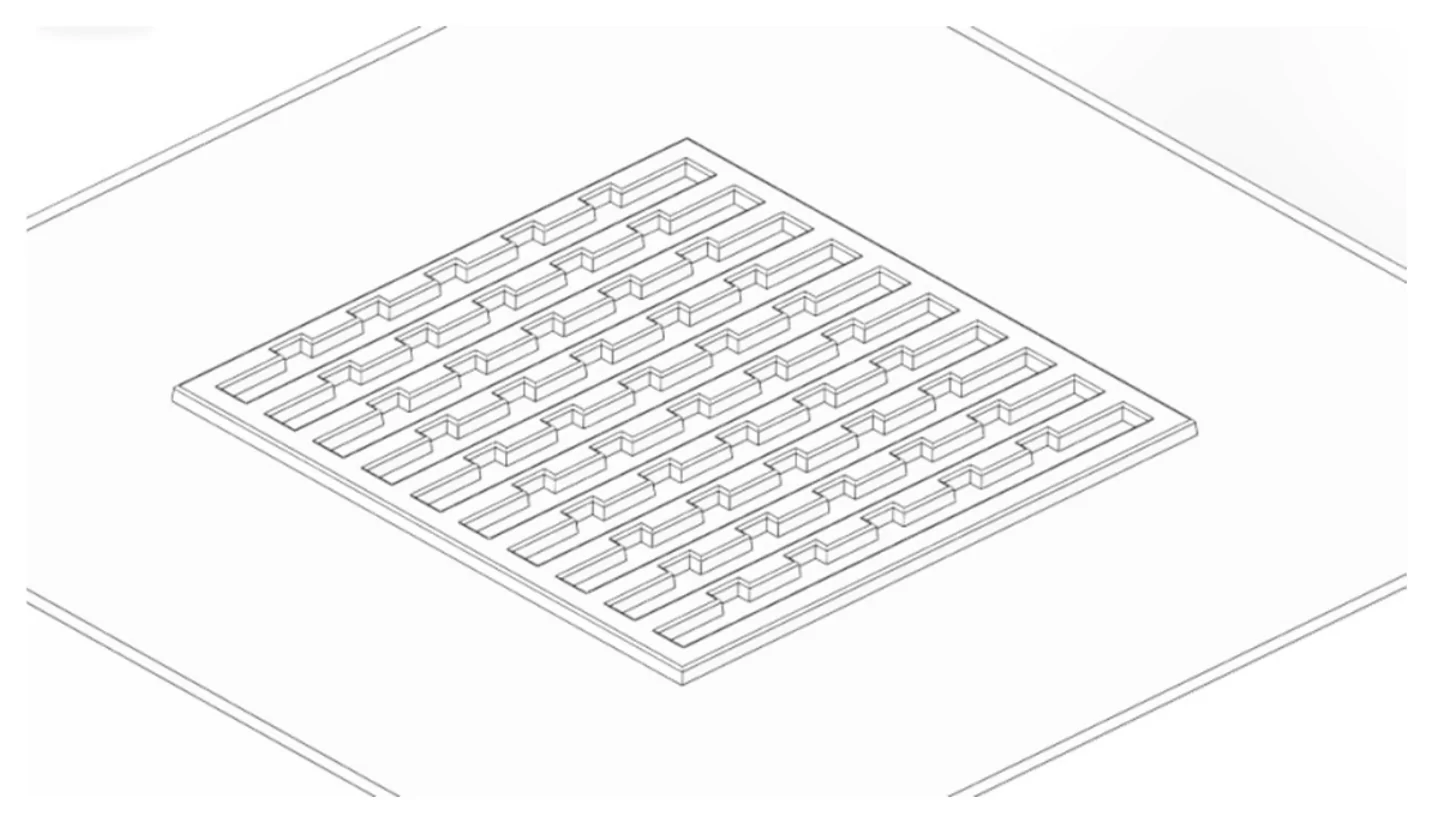
Schematic of the punch containing repeated local side-boss features within the parallel-channel region.

**Figure 2:**
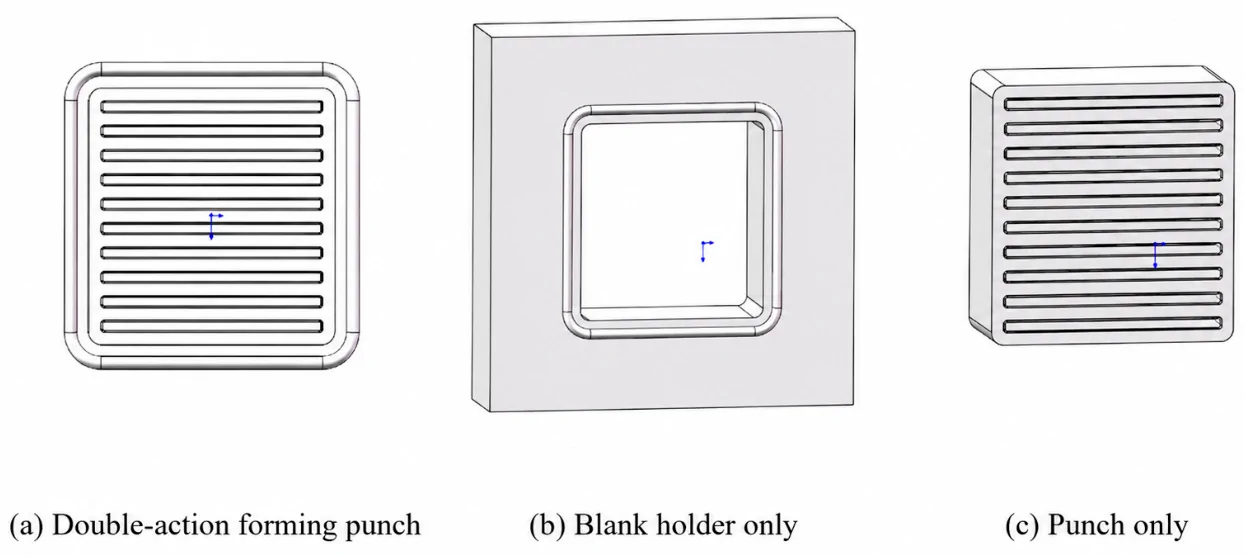
Simplified die of the metallic bipolar plate.

**Figure 3:**
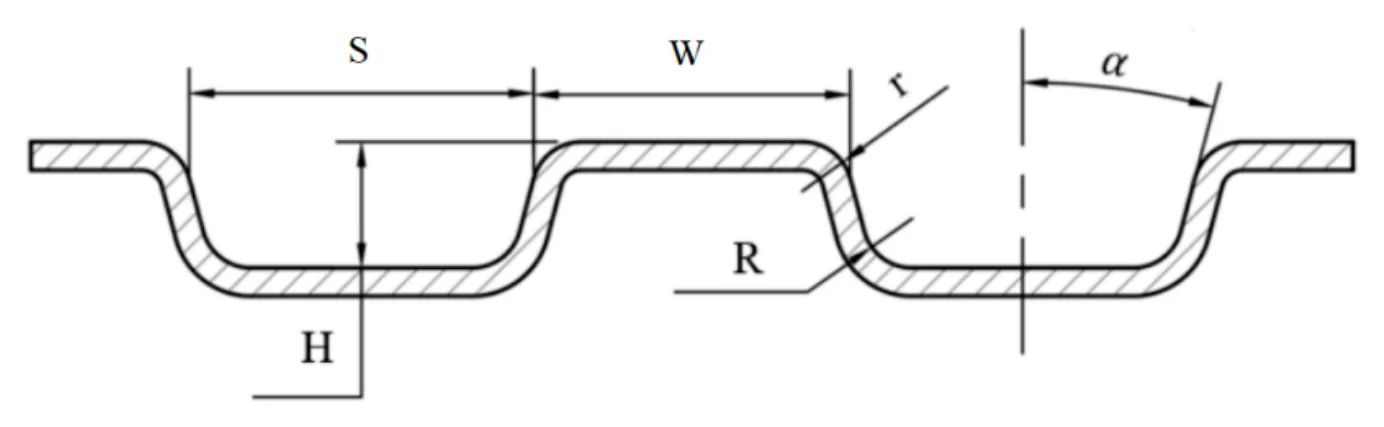
Cross-section of the metallic bipolar-plate flow channel.

The metallic bipolar plate and stamping tools were created in SolidWorks 2022. The punch geometry and its repeated local side-boss features are illustrated in Figure 1, while Figure 2 presents the simplified stamping assembly. The flow-field area was 21 mm × 20 mm, and the blank size was 40 mm × 40 mm with a thickness of 0.1 mm. From top to bottom, the tooling components were the punch, binder, blank, and die. The flow-channel cross-section and geometric definitions are provided in Figure 3, where S is the channel width, W is the rib width, H is the channel depth, R is the inner fillet radius, r is the outer fillet radius, and α is the draft angle.

The friction coefficient and blank-holder force were fixed at 0.125 and 1800 N. The basic dimensions were selected to provide an engineering-relevant metallic bipolar-plate forming scale. The blank thickness was 0.10 mm, the nominal channel width and rib width were both 1.00 mm, the channel depth was 0.50 mm, and the active flow-field region was 21 mm × 20 mm. Ultra-thin stainless-steel sheets near 0.1 mm and channel widths on the order of 1 mm are commonly investigated in metallic bipolar-plate forming studies [28,30,31,35]. These dimensions therefore serve as the fixed baseline for evaluating the additional forming demand created by the side-bosses.

The parameter matrix connects performance-oriented geometry selection with systematic formability assessment. The electrochemical basis for selecting five repeated side-boss sets with a height of 0.45 mm is presented in Section 3.1, whereas the remaining configurations provide controlled comparisons of feature density, protrusion severity, and punch speed response.

The number of repeated side-boss sets was varied as M = 0, 1, 2, 3, 4, and 5, where M = 0 denotes the unmodified parallel-channel baseline. The side-boss height H_b_, defined as the transverse protrusion distance from the channel sidewall, was varied as 0, 0.15, 0.30, 0.45, 0.60, and 0.75 mm. Relative to the nominal channel width S = 1.0 mm, these values correspond to H_b_/S = 0–0.75. The uniform 0.15 mm increment spans mild, moderate, performance-oriented, and high-severity protrusions. The 0.60 and 0.75 mm cases extend the range toward severe deformation and reveal the onset of rapid forming-margin reduction. The complete 40 mm × 40 mm blank was modelled because global material draw-in, binder restraint, interactions among adjacent boss sets, and non-periodic flow-field edges and corners all contribute to the forming response. This parameter space systematically links the performance-oriented reference geometry with its manufacturability.

### 2.2. Material Model and Stamping Conditions

**Figure 4:**
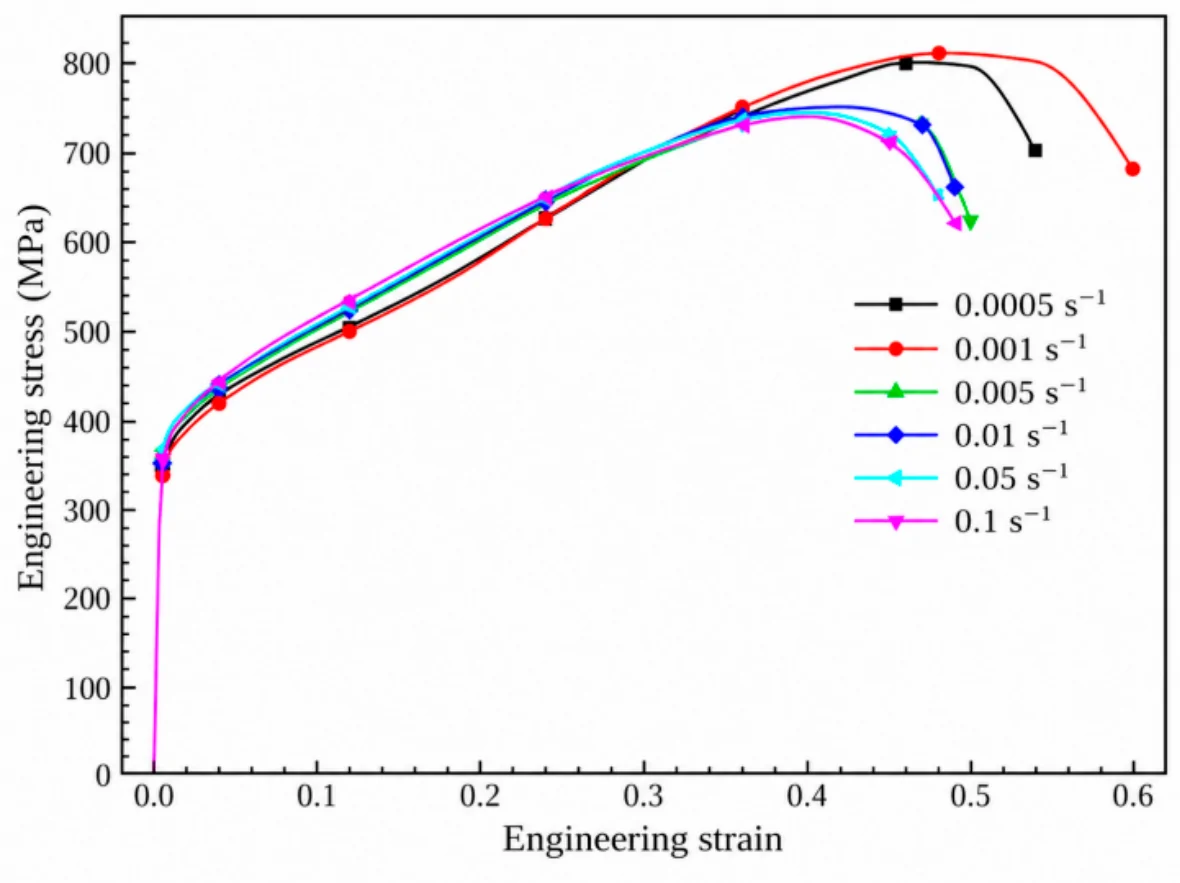
Literature-derived engineering stress–strain curves of SUS304 at nominal strain rates of 0.0005–0.1 s^−1^ [45].

**Figure 5:**
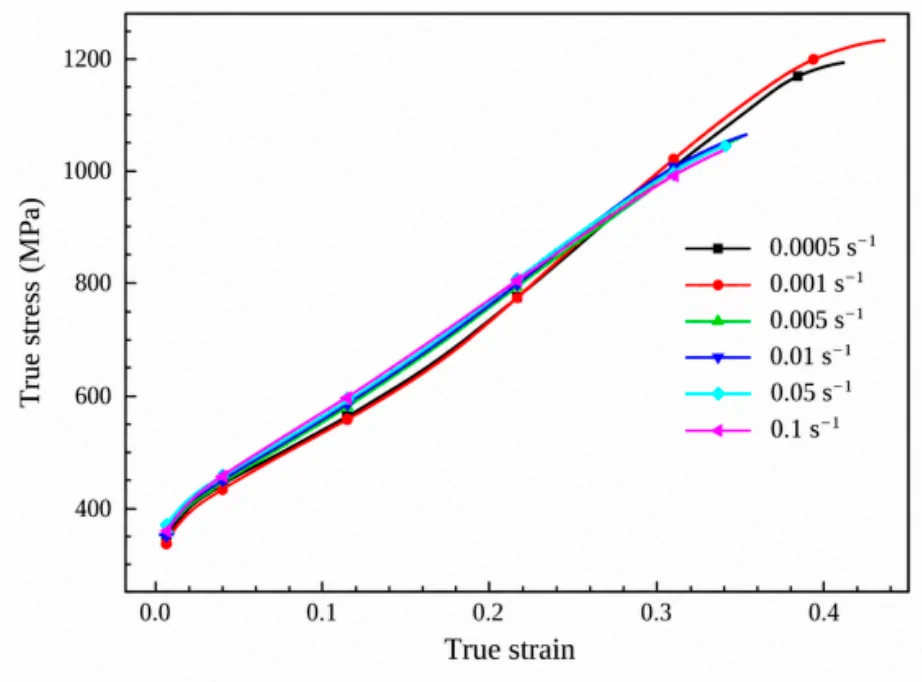
Literature-derived true stress–strain curves used in the SS304 material description [45].

The sheet blank was discretized using Belytschko–Tsay shell elements with five Gauss integration points through the thickness. The 0.1 mm SS304 blank was represented as a homogeneous, macroscopically isotropic elastic–plastic continuum. Plastic yielding was evaluated using an isotropic J_2_/von Mises yield surface. The elastic properties and initial yield strength are summarized in Table 1. The post-yield response was defined by piecewise tabulated true stress–plastic strain curves derived from the SUS304 tensile tests reported by Li et al. [45]. The engineering and corresponding true stress–strain curves are shown in Figure 4 and Figure 5.

The tabulated true stress–plastic strain curves govern the post-yield response, while *n* = 0.502 is used in the built-in forming-limit curve estimator. The curves span nominal tensile strain rates of 0.0005–0.1 s^−1^ [45], and recent SUS304/AISI 304 studies further confirm the rate-dependent strengthening and dynamic deformation characteristics of the alloy [46,47,48]. Punch speed is prescribed as the tool kinematic condition, and the resulting local strain-rate field evolves with geometry, contact sequence, and deformation history. The value r = 1.0 provides a consistent isotropic reference for all cases, ensuring that the observed differences arise from the investigated geometric and process variables. The influence of plastic anisotropy on stainless-steel foil microformability is documented in Ref. [49].

The material, contact, restraint, and tooling settings were maintained consistently throughout the parameter matrix, providing a common basis for isolating the effects of side-boss number, side-boss height, and punch speed.

The tools were meshed primarily with quadrilateral elements. A graded local-refinement strategy was used for the blank: the smallest in-plane element size of 0.025 mm was assigned to side-boss roots, root chamfers, boss tops, channel-bottom fillets, sidewall transitions, and flow-field corners, whereas the mesh was gradually coarsened to a maximum of 0.4 mm in remote flange regions. The contact friction coefficient between the tools and blank was fixed at 0.125. The reference channel geometry was S = W = 1 mm, channel depth H = 0.5 mm, inner and outer fillet radii R = r = 0.1 mm, and draft angle α = 5°.

The forming sequence comprised Closing and Drawing. During Closing, the binder contacted the blank through a penalty contact formulation with a penalty factor of 0.15 and a closing gap of 0.11 mm. During Drawing, the punch advanced until final closure, with the same nominal gap. The binder and punch motion histories were prescribed using the Dynaform default velocity curve. The blank-holder force was calculated from F_BH_ = Aq, where A is the binder-projected blank area and q is the unit blank-holder pressure.

(1) $$F_{BH}=Aq$$

For the present blank, A was approximately 600 mm^2^ and q was set to 3 MPa, giving F_BH_ = 1800 N. The selected friction coefficient μ = 0.125 and blank-holder force F_BH_ = 1800 N establish a consistent contact and restraint baseline for isolating the effects of side-boss geometry and punch speed. Their coupled influence on material draw-in is discussed in Section 3.5. The numerical model and fixed analysis conditions are summarized in Table 2.

### 2.3. Formability Evaluation Framework

**Figure 6:**
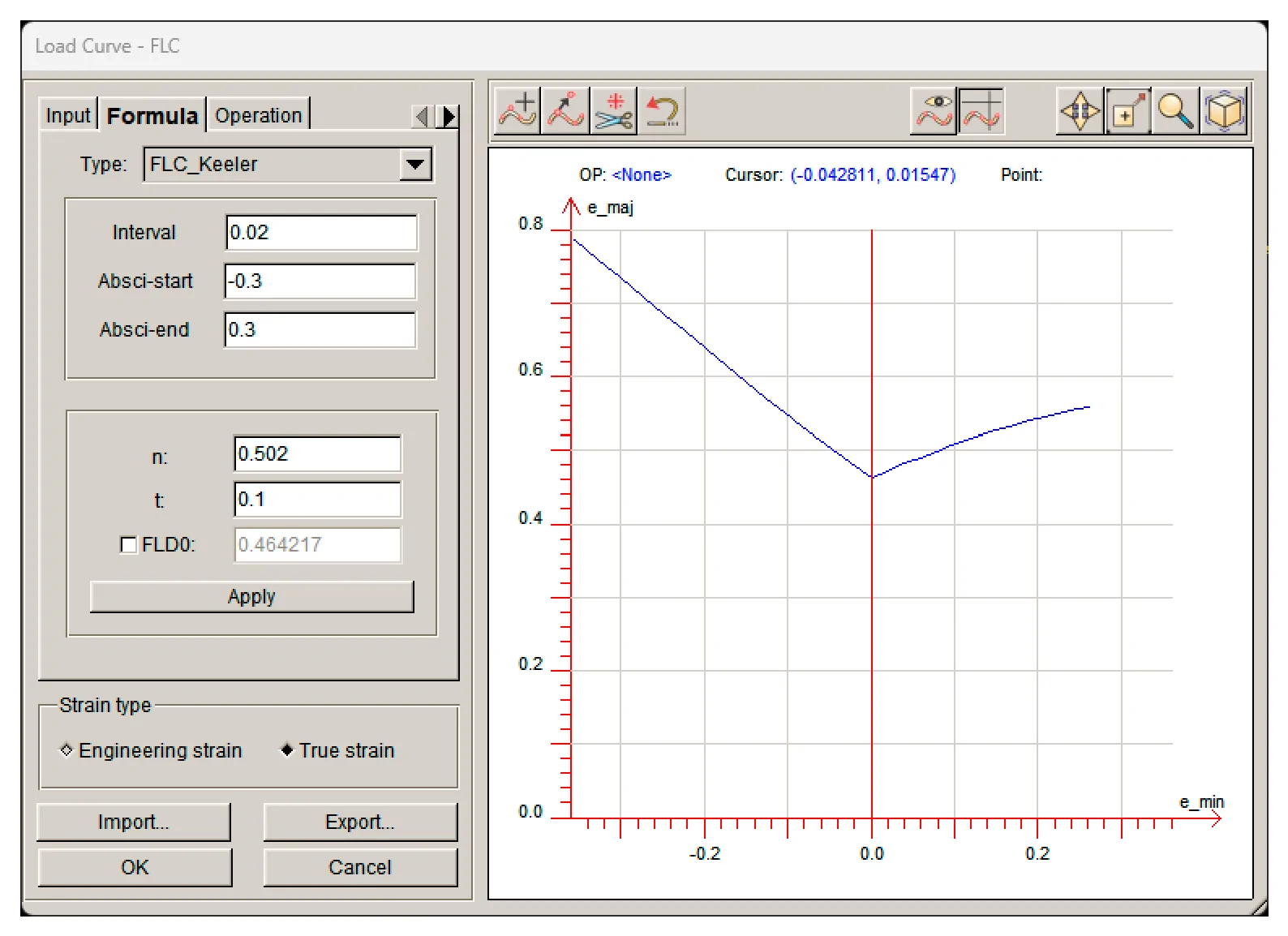
Forming−limit curve (FLC) in Dynaform.

The Dynaform forming-limit diagram maps the calculated major and minor in-plane strains relative to a material forming-limit curve. The FLC may be defined from measured Nakajima or Marciniak strain data [50] or generated using the software’s empirical estimation module. For the present comparative matrix, the curve was generated from the adopted sheet thickness, *n* = 0.502, and r = 1.0, as shown in Figure 6.

The calculated major–minor strain states were compared with the estimated FLC to characterize localized-necking initiation. The resulting categories are formulated as forming-risk states and provide a consistent strain-limit reference across the complete parameter matrix.

The Dynaform FLD maps also contain compressive or instability-related regions associated with wrinkling tendency. Tensile local-necking and compressive wrinkling are therefore treated as distinct forming responses. The operational categories used in the Results and Discussion are defined in Table 3. The state assigned to each case considers the most severe spatially meaningful region together with its continuity and corresponding thickness localization; clustered points at a consistent geometric concentration carry greater weight than an isolated point.

Accordingly, the estimated FLD is evaluated together with maximum thinning ratio, thickness-distribution contours, and the locations of high-curvature deformation. This combined assessment links strain path, thinning magnitude, and geometric location.

### 2.4. Mesh Convergence and Model Credibility

Mesh convergence was assessed before the parameter comparisons using identical material, contact, loading, and solver settings while varying only the local minimum element size. Local sizes of 0.100, 0.075, 0.050, and 0.025 mm were evaluated, and the resulting maximum-thinning response was used to select the final discretization.

**Figure 7:**
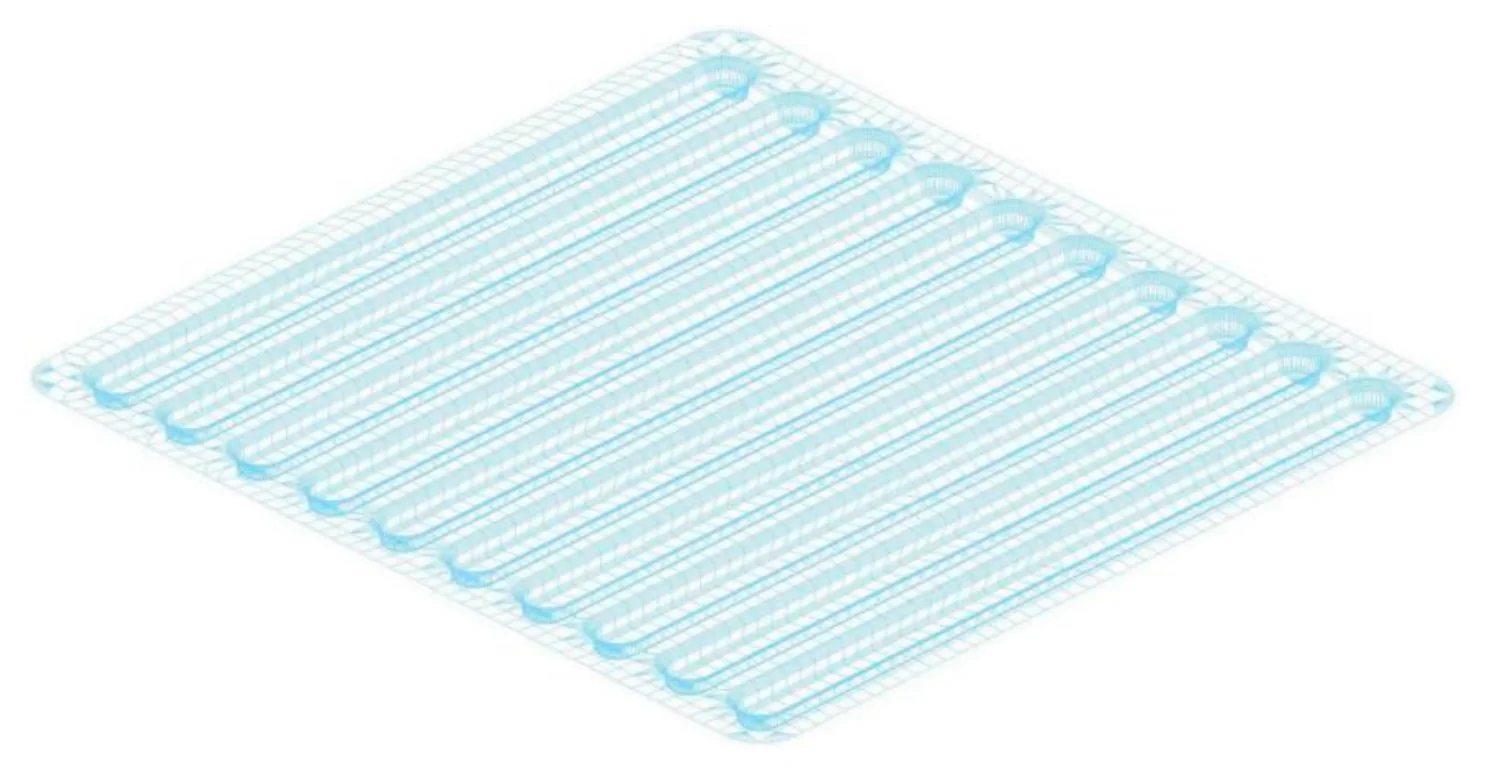
Locally refined mesh used in the forming simulations.

The graded local-refinement strategy used to concentrate resolution in the active forming region is illustrated in Figure 7. The finest elements were assigned to side-boss roots, root chamfers, boss tops, channel-bottom fillets, sidewall transitions, and flow-field corners, where curvature and strain gradients are highest, while remote flange regions were progressively coarsened. Because the reference fillet radius is 0.1 mm, a local size of 0.025 mm provides approximately four elements over a characteristic 0.1 mm fillet length, compared with approximately one element for the 0.100 mm mesh.

Mesh difference was evaluated using the 0.025 mm result as the reference:

(2) $$\delta_{h}=\frac{\left|\eta_{h}-\eta_{0.025}\right|}{\eta_{0.025}}\;\times \;100\%$$

where η_h_ is the maximum thinning ratio obtained using local element size h. Relative to the 0.025 mm reference, the 0.050 and 0.075 mm meshes differ by approximately 1.07% and 0.70%, respectively, whereas the 0.100 mm mesh differs by approximately 4.81%. The response stabilizes for the three finer meshes, while the 0.100 mm mesh under-resolves the high-curvature deformation.

Among the converged candidates, the 0.025 mm local mesh was selected for the final parameter study. A 0.050 mm mesh is adequate for preliminary screening, but it provides only approximately two elements across a 0.1 mm fillet. The 0.025 mm mesh provides approximately four elements over the same characteristic length and is therefore the most conservative choice for resolving the boss root, fillet, boss top, and corner locations that govern the conclusions.

**Figure 8:**
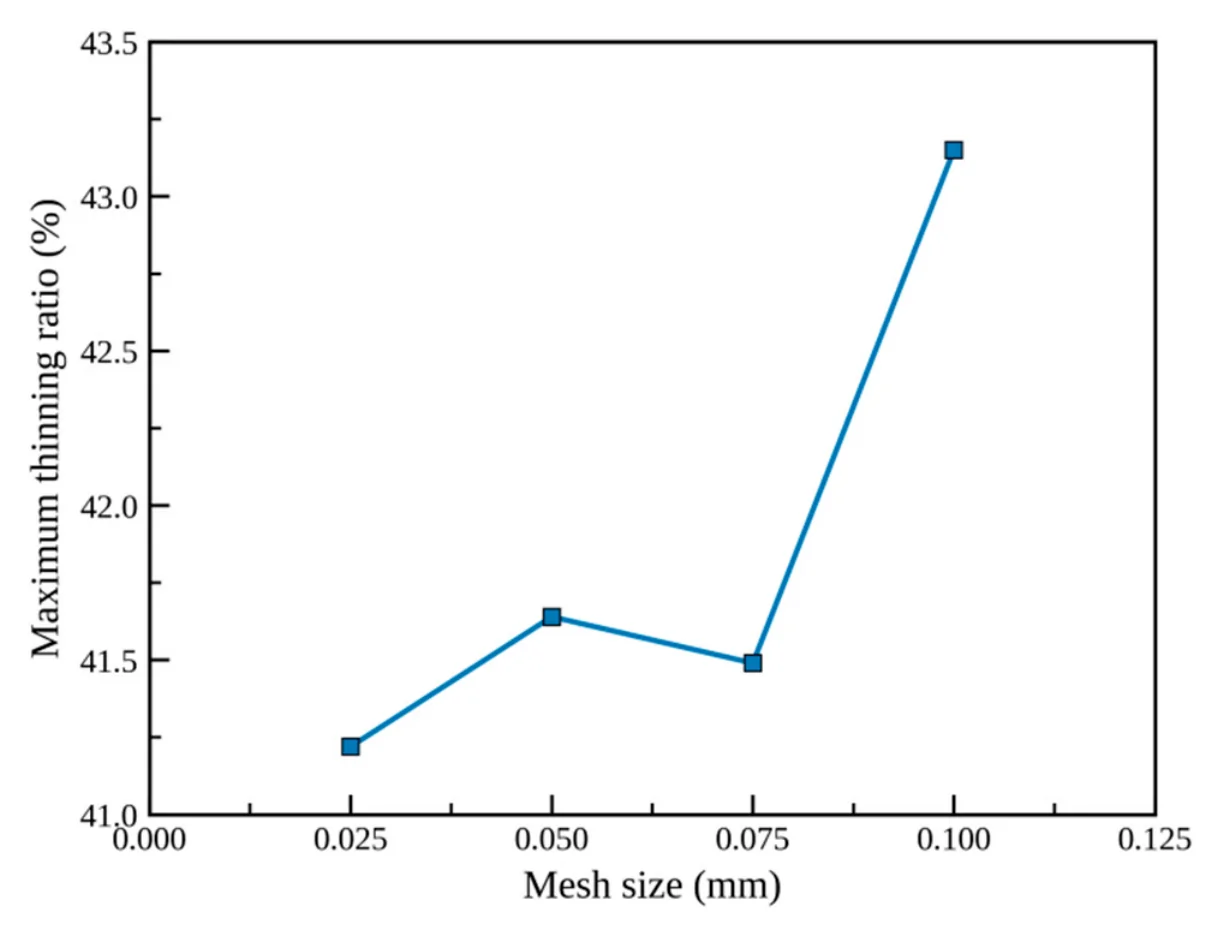
Maximum thinning ratio obtained with different local mesh sizes.

The convergence trend is summarized graphically in Figure 8 and quantitatively in Table 4. The small response change relative to the coarser converged meshes, together with the improved geometric resolution, supports retention of the 0.025 mm local mesh. This selection is based on both stabilization of maximum thinning and spatial fidelity of the critical-location pattern.

In addition to mesh convergence, model credibility was evaluated against published experimental–numerical investigations using three common indicators: the order of thinning magnitude, the dominant strain-path response, and the localization of severe deformation at sheet–tool contact transitions.

Hu et al. [30] showed that Dynaform reproduced the principal thickness and defect trends of stamped SS304 bipolar plates, including a comparatively stable intermediate-speed response. Talebi-Ghadikolaee et al. [13] reported thickness and force-displacement errors of approximately 4.76% and 3.85%, respectively, together with a 33.45% critical thinning level dominated by plane-strain tension. These results provide a quantitative reference for the present thinning range and speed-dependent response.

Zhang et al. [17] located severe thinning and local-necking initiation at high-curvature sheet–tool contact regions in approximately 0.1 mm stainless-steel sheets. Acar et al. [18] likewise demonstrated the coupled influence of blank-holder restraint, forming velocity, contact transition, and springback. The boss roots, boss tops, local fillets, and flow-field corners identified here exhibit the same geometry-controlled localization mechanism.

Together, the mesh-convergence trend and the published experimental–numerical evidence establish consistent response magnitude, strain-path interpretation, and critical-location patterns for the present comparative analysis. A comparison of the present results with published experimental–numerical stamping studies is provided in Table 5.

## 3. Performance and Formability Results and Discussion

The side-boss flow field must satisfy both electrochemical transport requirements and stamping-formability constraints. This section first establishes the performance basis for the reference geometry and then quantifies the effects of side-boss number, side-boss height, and punch speed on thinning, strain-path evolution, and critical-region migration.

All stamping cases are compared under the common material, contact, restraint, and tooling conditions summarized in Table 2. The estimated-FLD states follow the definitions in Table 3 and are interpreted jointly with thickness distribution and maximum thinning ratio.

### 3.1. Electrochemical Performance and Reference-Geometry Selection

The side-boss geometry was selected using a three-dimensional PEMFC performance model that connects the flow-field transport response with the subsequent manufacturability assessment.

The screening model represented the anode and cathode flow channels, gas-diffusion layers, catalyst layers, proton-exchange membrane, and bipolar-plate/current-collector domains. A three-dimensional, steady-state multiphysics formulation coupled mass and momentum conservation, reactant-species and water transport, electronic and protonic charge conservation, and Butler–Volmer electrochemical kinetics. Gas flow was treated as laminar, the gas mixture as ideal, the porous layers as homogeneous continua, and gravity was neglected. The governing framework and transport assumptions follow established PEMFC flow-field modelling practice [36,37,38,39,40,41,42,43,44].

Identical material properties, operating temperature, inlet composition and flow conditions, outlet pressure, electrochemical parameters, solver tolerances, and mesh strategy were applied to all candidate geometries; only the number of side-boss sets was changed at H_b_ = 0.45 mm. Uniform inlet conditions, pressure outlets, no-slip solid walls, and zero normal species/charge fluxes at impermeable external boundaries were prescribed. Cell voltage was varied to generate the polarization and power-density curves, and the mesh was locally refined through the membrane electrode assembly and around the channel/side-boss transitions. The oxygen-distribution uniformity index at the cathode channel/GDL interface was evaluated as $\gamma_{O_{2}}=\;1-\frac{\sigma_{O_{2}}}{\mu_{O_{2}}}$, where μ_O2_ and σ_O2_ are the area-weighted mean and standard deviation of oxygen mass fraction, respectively.

**Figure 9:**
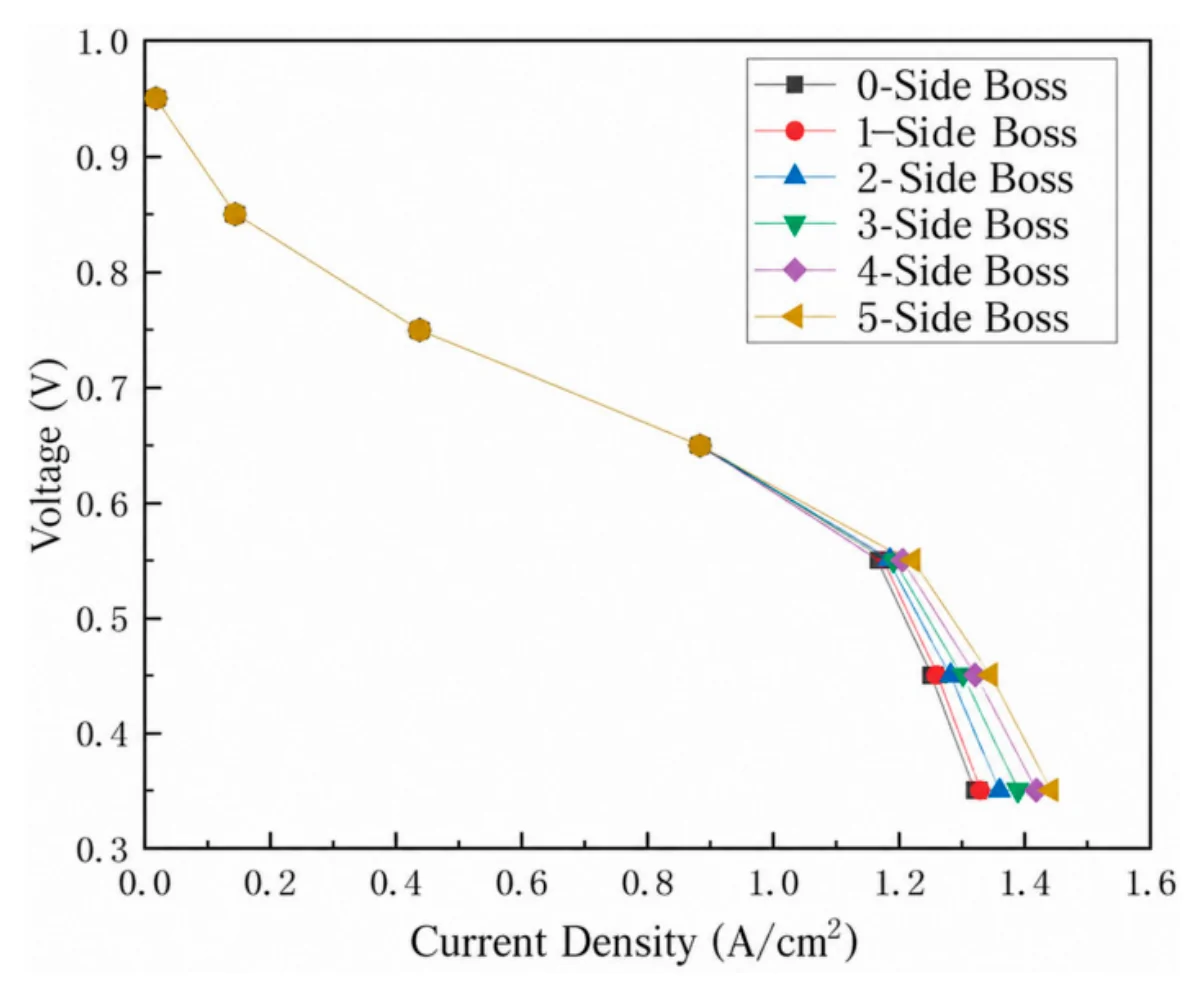
Polarization curves for different numbers of side-boss sets.

**Figure 10:**
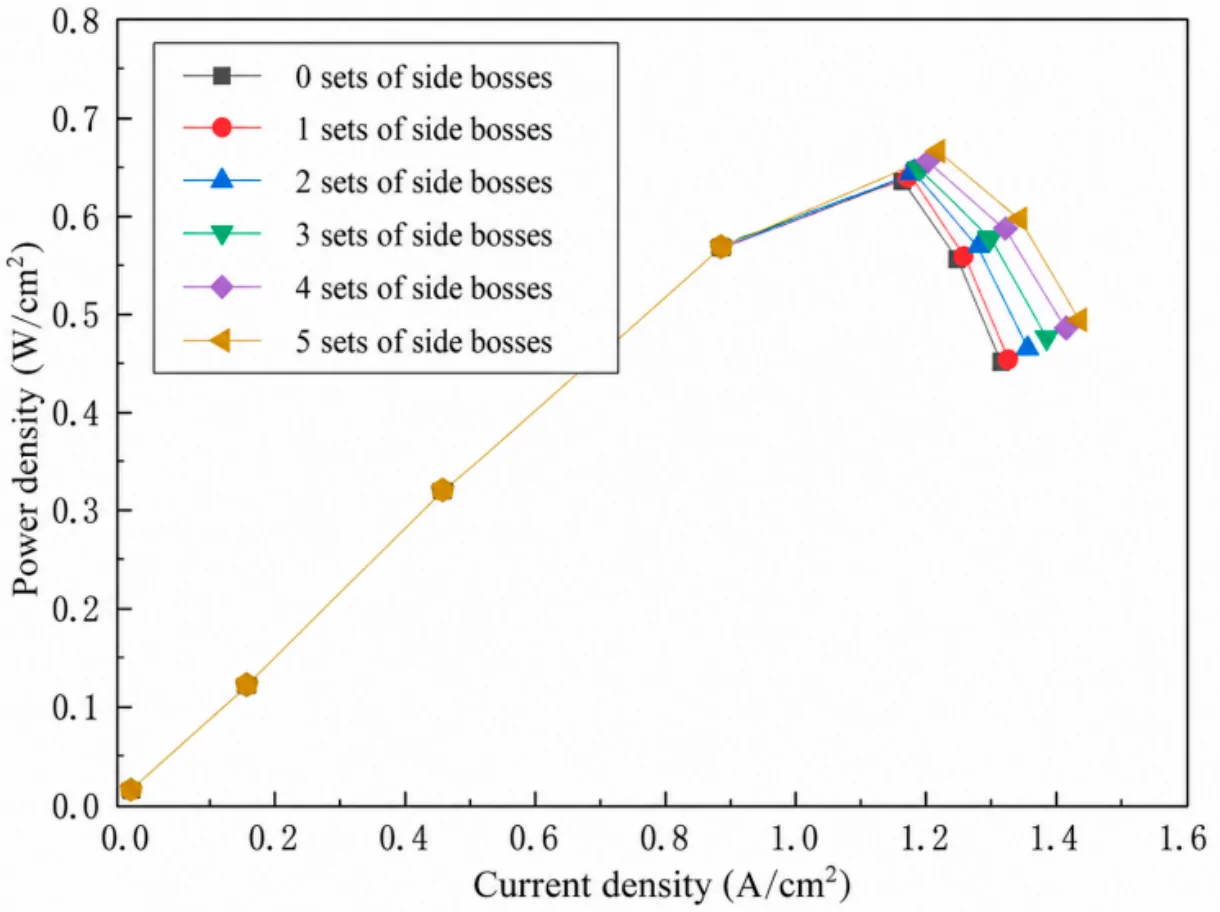
Power-density curves for different numbers of side-boss sets.

The polarization and power-density responses in Figure 9 and Figure 10 identify M = 5 and H_b_ = 0.45 mm as the performance-oriented reference geometry for the stamping study.

**Figure 11:**
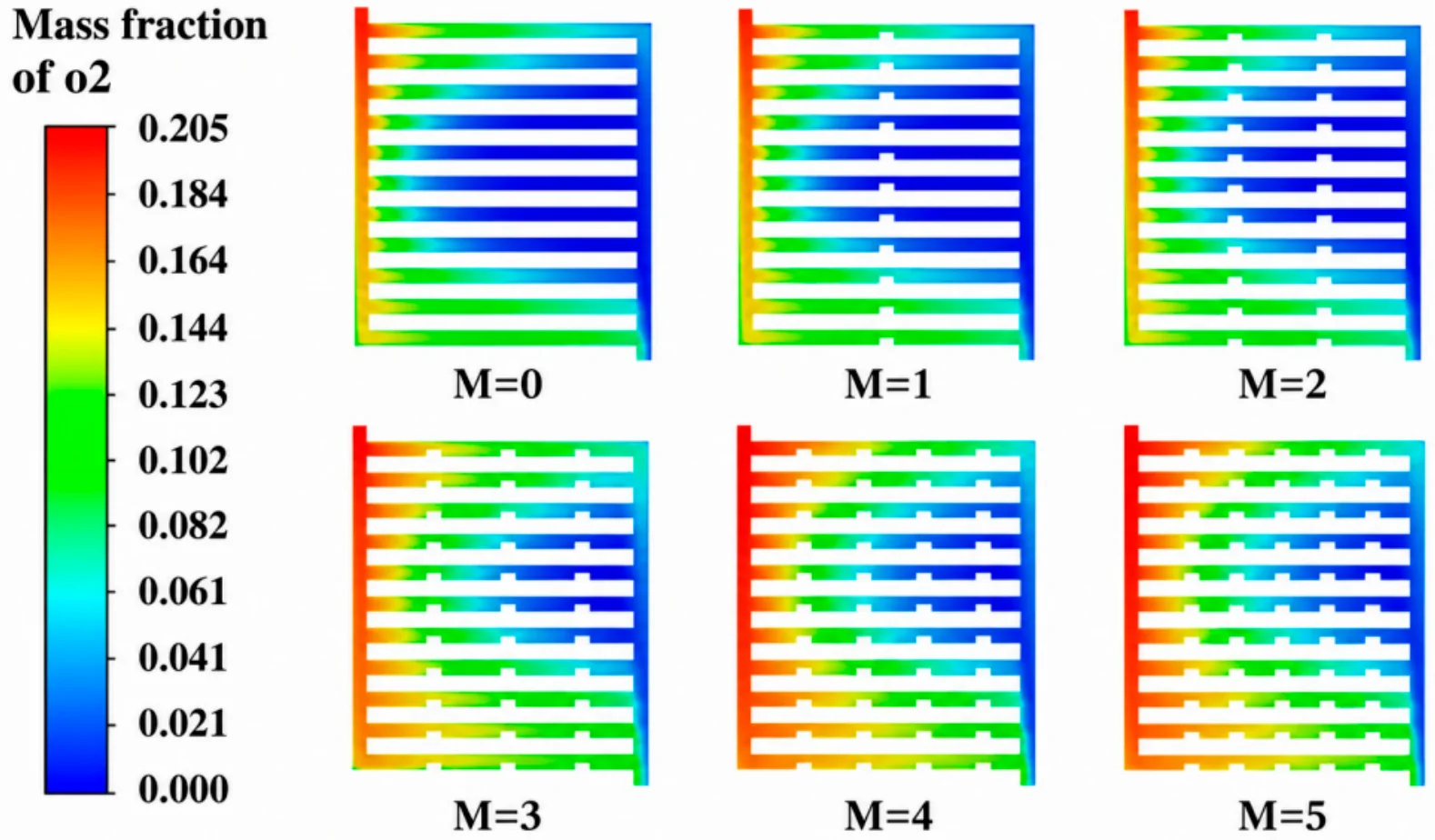
Oxygen mass fraction at the cathode channel/GDL interface.

**Figure 12:**
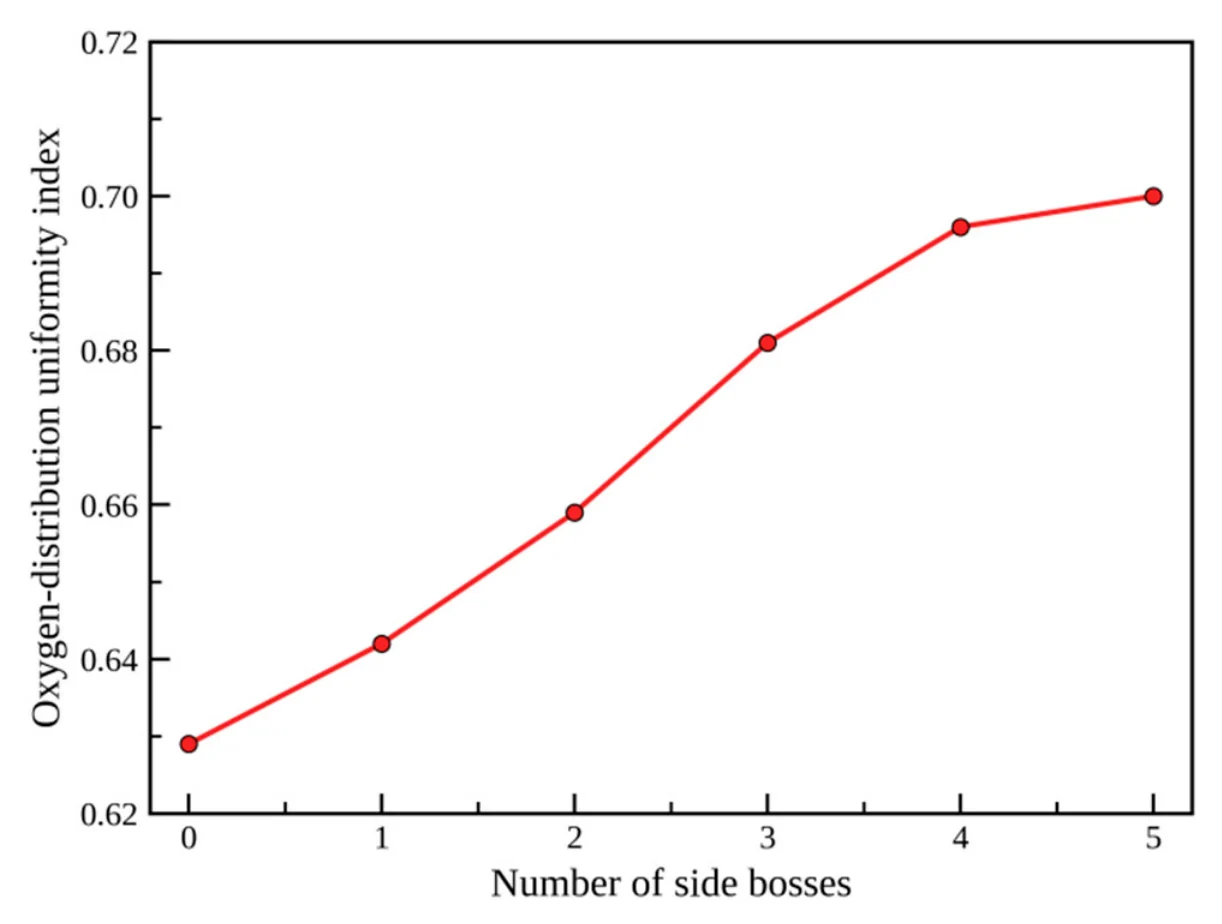
Oxygen-distribution uniformity index at the cathode channel/GDL interface.

From the polarization curves and power density curves, the introduction of side-bosses helps improve the output performance of the parallel flow field. The curves in Figure 9 and Figure 10 indicate that the power density reaches its peak around 0.55 V. When the number of side-bosses is 5 and the height is 0.45 mm, the current density and power density show the best performance. Compared with the parallel flow field without side-bosses, the peak power density under this condition increases by 3.02%, and the oxygen distribution uniformity index increases by 11.61%. This result indicates that side-bosses do not merely increase geometric complexity; within a certain parameter range, they indeed demonstrate potential for performance improvement. To examine the transport mechanism underlying this gain, Figure 11 and Figure 12 compare the cathode channel/GDL oxygen mass-fraction field and its distribution-uniformity index.

In terms of oxygen distribution, the parallel flow field presents an oxygen-deficient zone in the middle region near the outlet. The side-boss arrangement redirects gas flow and promotes oxygen transport toward the downstream channel and gas diffusion layer (GDL). As shown in Figure 11 and Figure 12, the low-oxygen region progressively contracts and the oxygen mass-fraction distribution becomes more uniform as the number of side-boss sets increases. With five sets, the oxygen-distribution uniformity improves by 11.61% relative to the conventional parallel flow field. Together with the 3.02% increase in peak power density, this result establishes the five-set geometry as the performance-oriented reference for the manufacturability analysis.

These results establish the electrochemical value of the five-set, 0.45 mm side-boss geometry. The following sections quantify its forming response and compare the effects of boss number, boss height, and punch speed on manufacturing feasibility.

### 3.2. Effect of Side-Boss Number on Formability

The number of repeated side-boss sets was varied from 0 to 5 while the height was fixed at 0.45 mm. The range spans a no-boss baseline and sparse-to-dense repeated layouts within the fixed 21 mm × 20 mm flow-field region without changing the principal channel count. The five-set case is retained because it was performance-motivated by the preliminary PEMFC simulations, whereas the remaining cases provide a controlled numerical screen of increasing local feature density.

**Figure 13:**
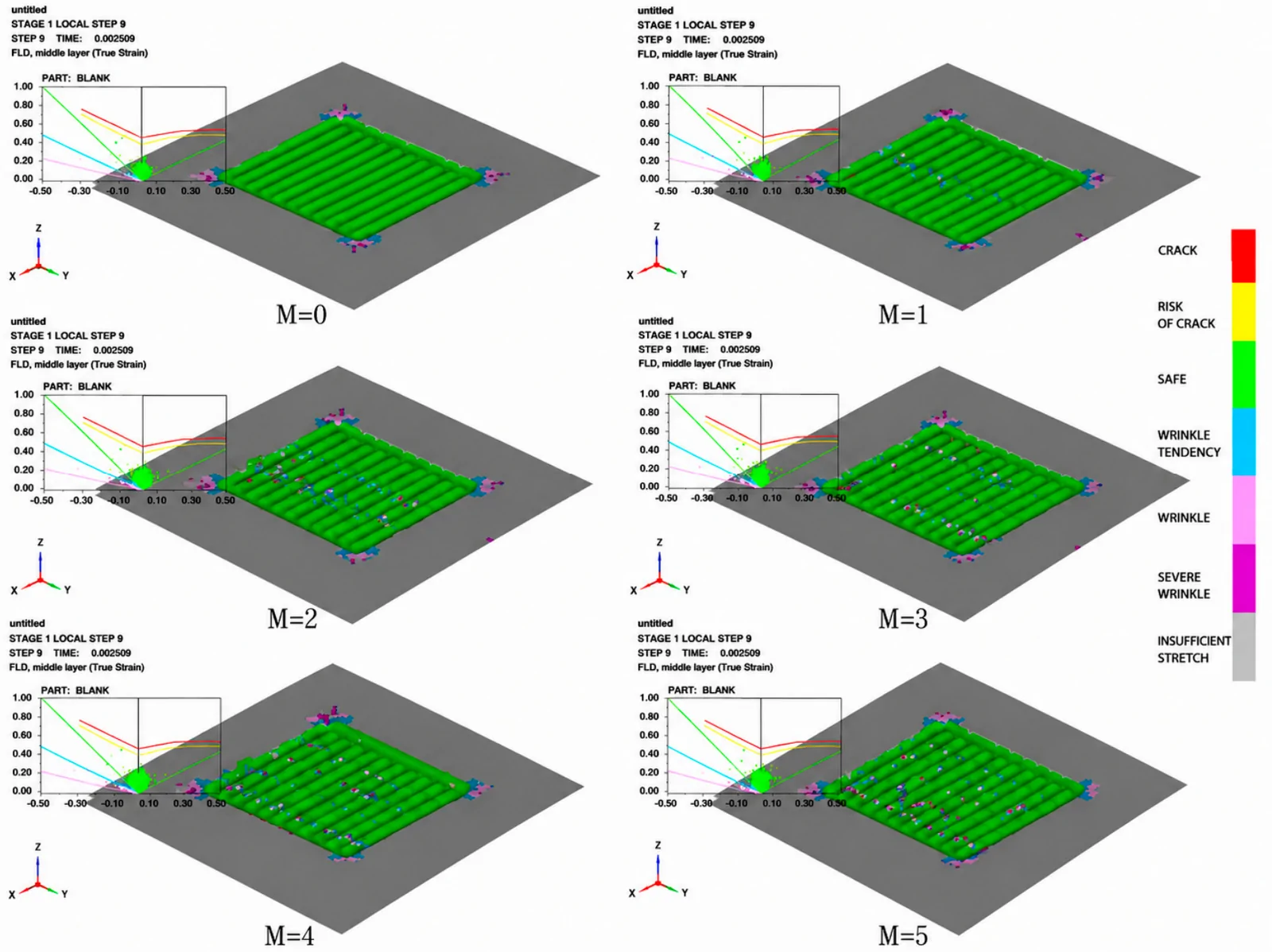
Estimated−FLD maps for different numbers of side-boss sets.

The location trend is consistent with published metallic bipolar-plate studies. Feng et al. [35] reported geometry-induced wrinkling near channel edges and raised features and cracking tendency near bottom fillets. Experimental–numerical investigations likewise identify tool-contact transitions and high-curvature regions as common thinning locations [13,17,26,30]. The present side-boss geometry introduces additional high-curvature root and top regions, which explains why the critical location differs from the centre of the unmodified channel. This geometry-induced shift in strain path is compared across the six cases in the estimated-FLD maps of Figure 13.

**Figure 14:**
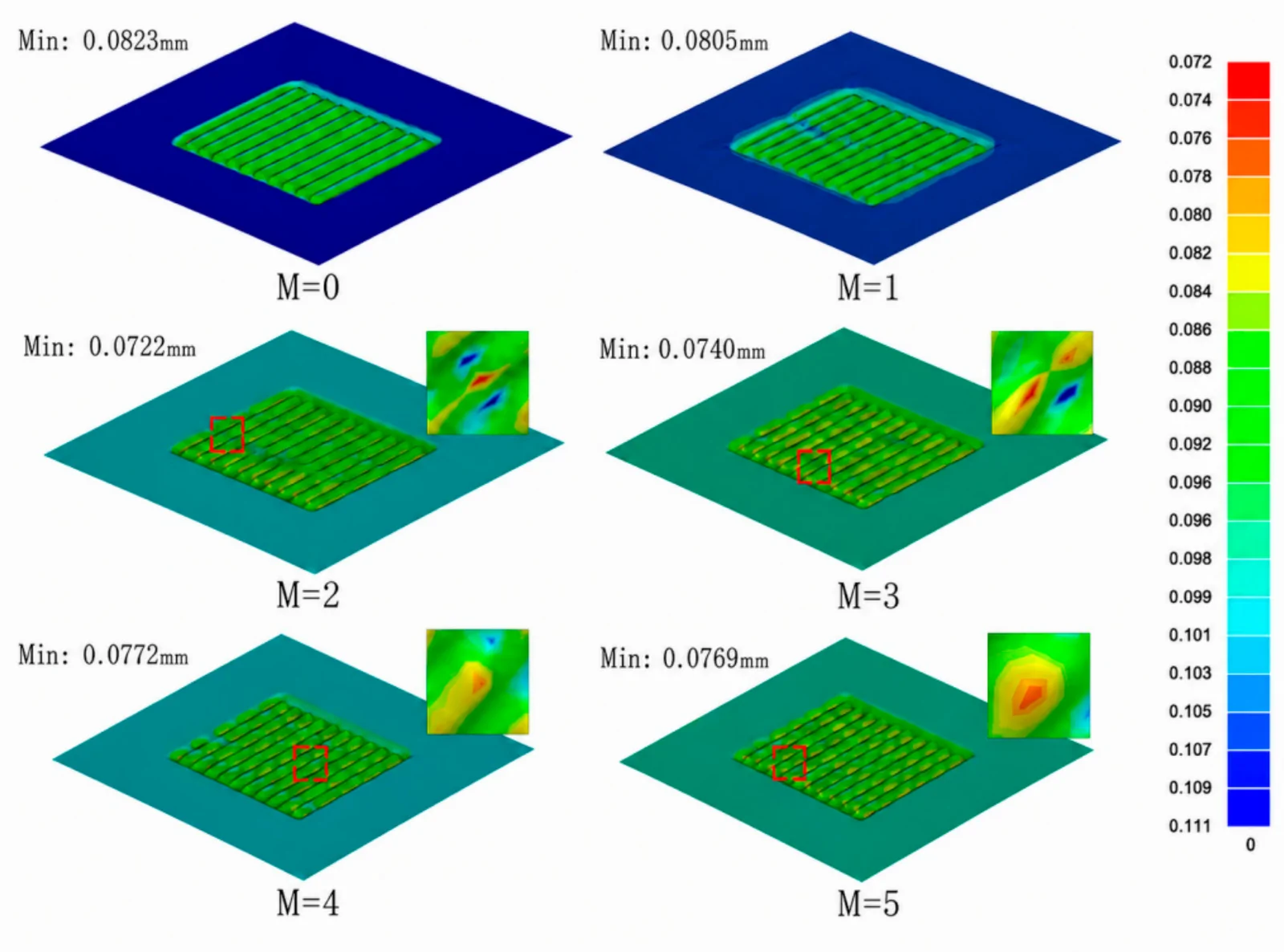
Thickness distributions for different numbers of side-boss sets.

**Figure 15:**
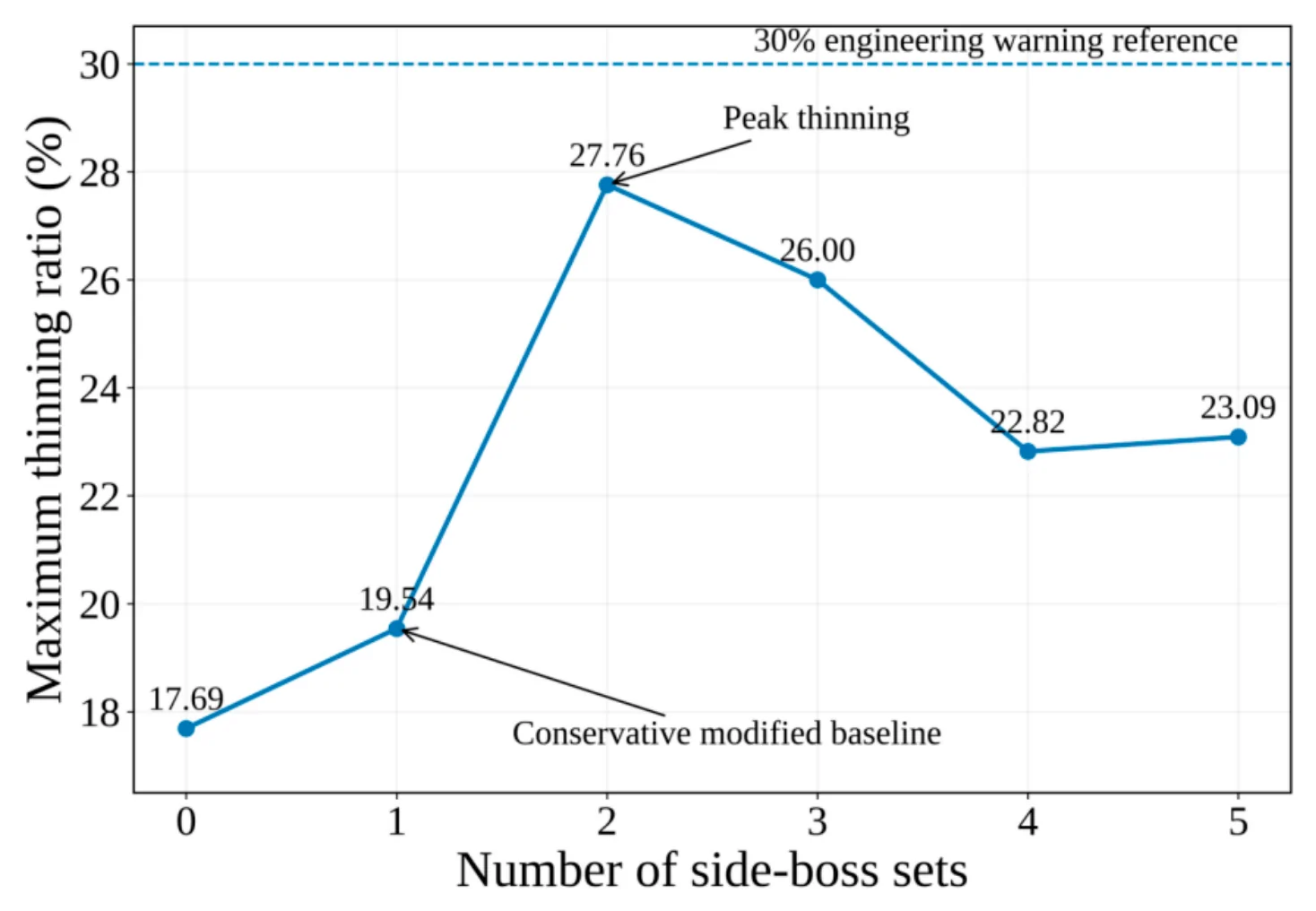
Maximum thinning ratio as a function of side-boss number.

The thickness fields and maximum-thinning response are compared in Figure 14 and Figure 15, respectively. The maximum thinning ratios are 17.69%, 19.54%, 27.76%, 26.00%, 22.82%, and 23.09% for 0–5 side-boss sets, respectively. The response is non-monotonic. The initial introduction of one and two sets disrupts the uniform material-flow path and concentrates deformation at boss root chamfers. At three and four sets, neighbouring deformation zones redistribute the flow to some extent, reducing the scalar peak. At five sets, broader high-risk regions develop near boss tops and the flow-field edge even though the maximum thinning remains below that of the two-set case. This difference confirms that maximum thinning and estimated-FLD spatial continuity must be evaluated together.

Side-boss number therefore exhibits a non-monotonic response. One set gives the lowest thinning among the modified cases and remains below the estimated FLC. The two-set case shows local-necking tendency at the boss root chamfer, the three- and four-set cases approach the estimated FLC, and the five-set case develops spatially broader high-risk regions near the boss tops and flow-field edge. The five-set configuration therefore represents a clear performance–formability trade-off.

### 3.3. Effect of Side-Boss Height on Formability

**Figure 16:**
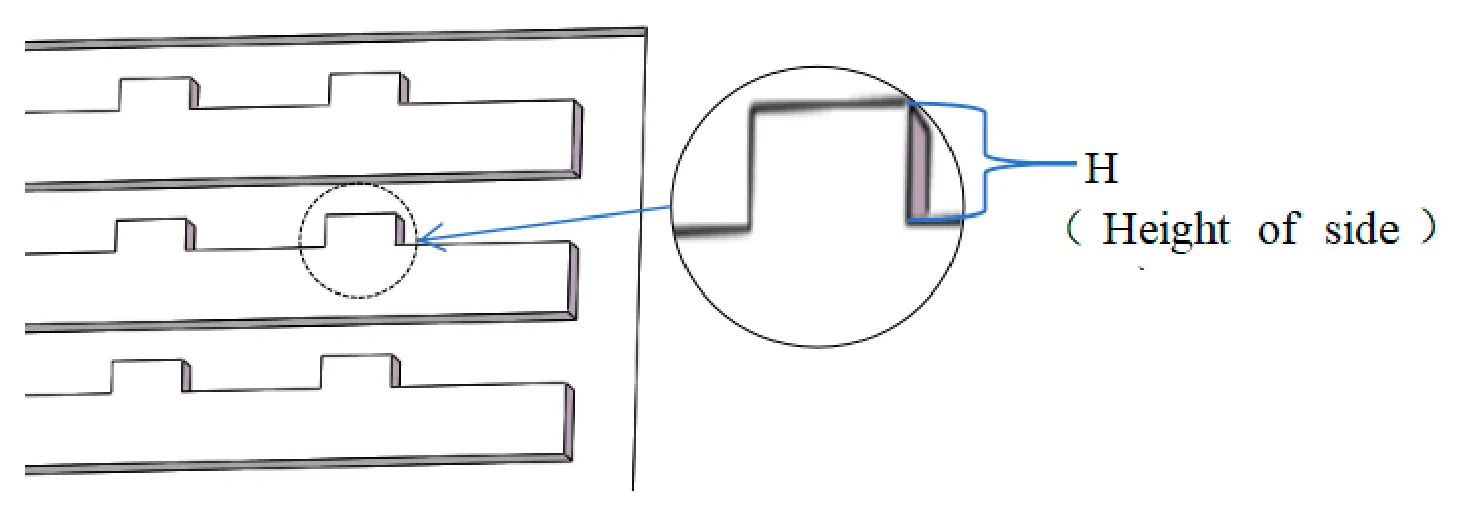
Definition of side-boss height H_b_.

**Figure 17:**
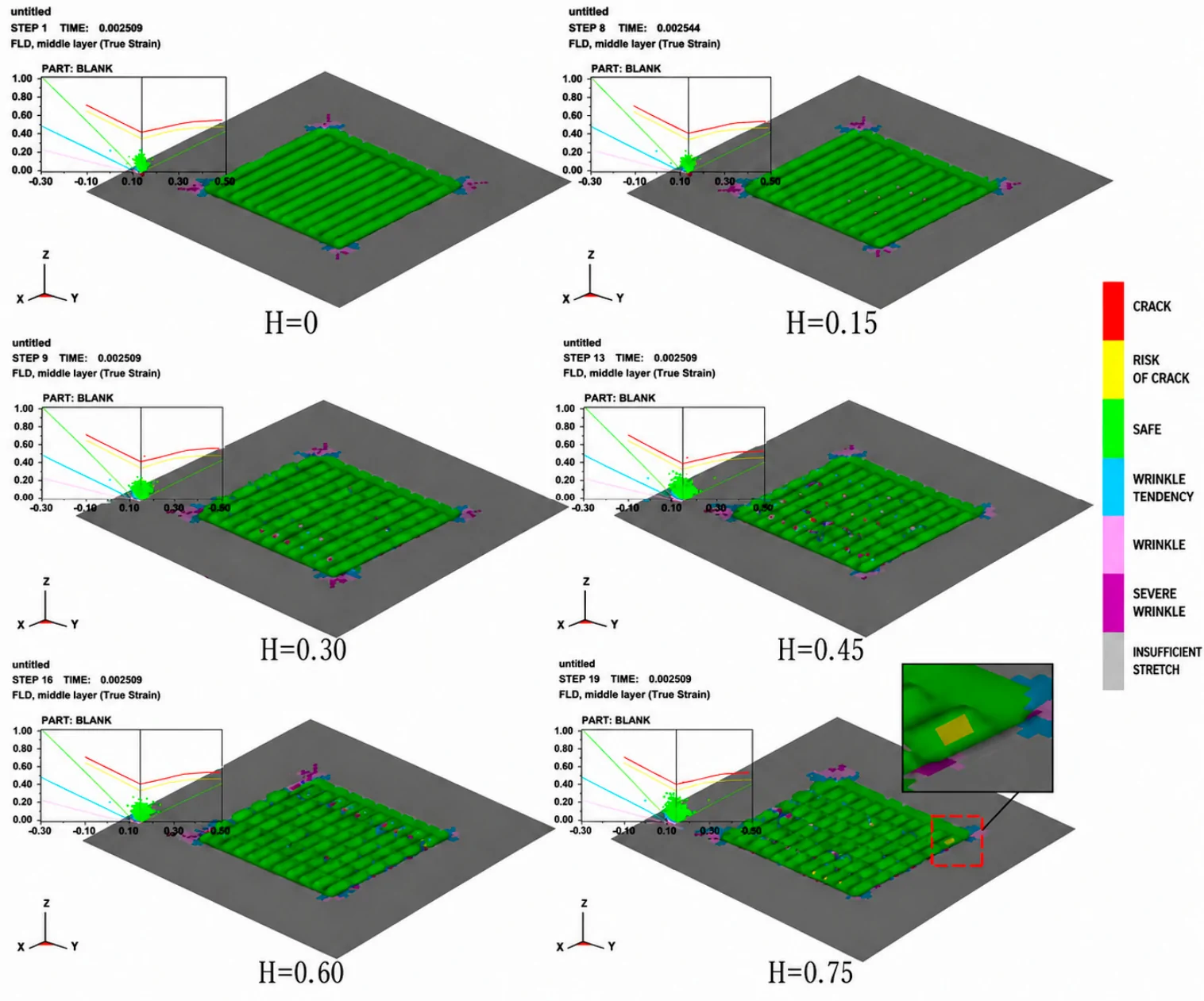
Estimated-FLD maps for different side-boss heights.

The height study used five side-boss sets to evaluate the performance-oriented configuration under progressively increasing forming severity. The geometric definition of H_b_ is given in Figure 16, whereas Figure 17 traces the corresponding evolution of the estimated-FLD response. Heights of 0, 0.15, 0.30, 0.45, 0.60, and 0.75 mm were selected at uniform 0.15 mm intervals. The upper values probe the high-severity range and reveal the transition from gradual to rapid loss of forming margin.

**Figure 18:**
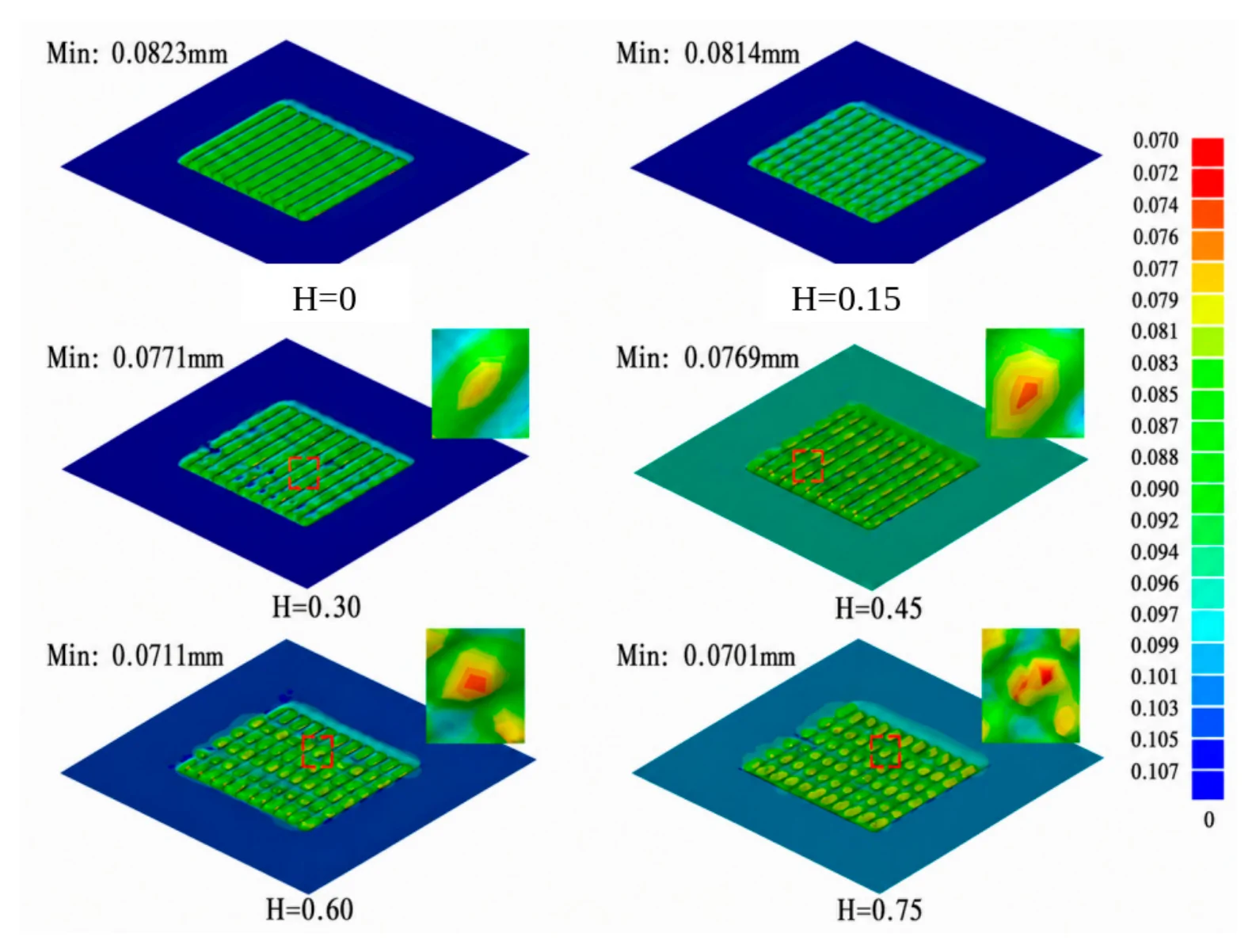
Thickness distributions for different side-boss heights.

**Figure 19:**
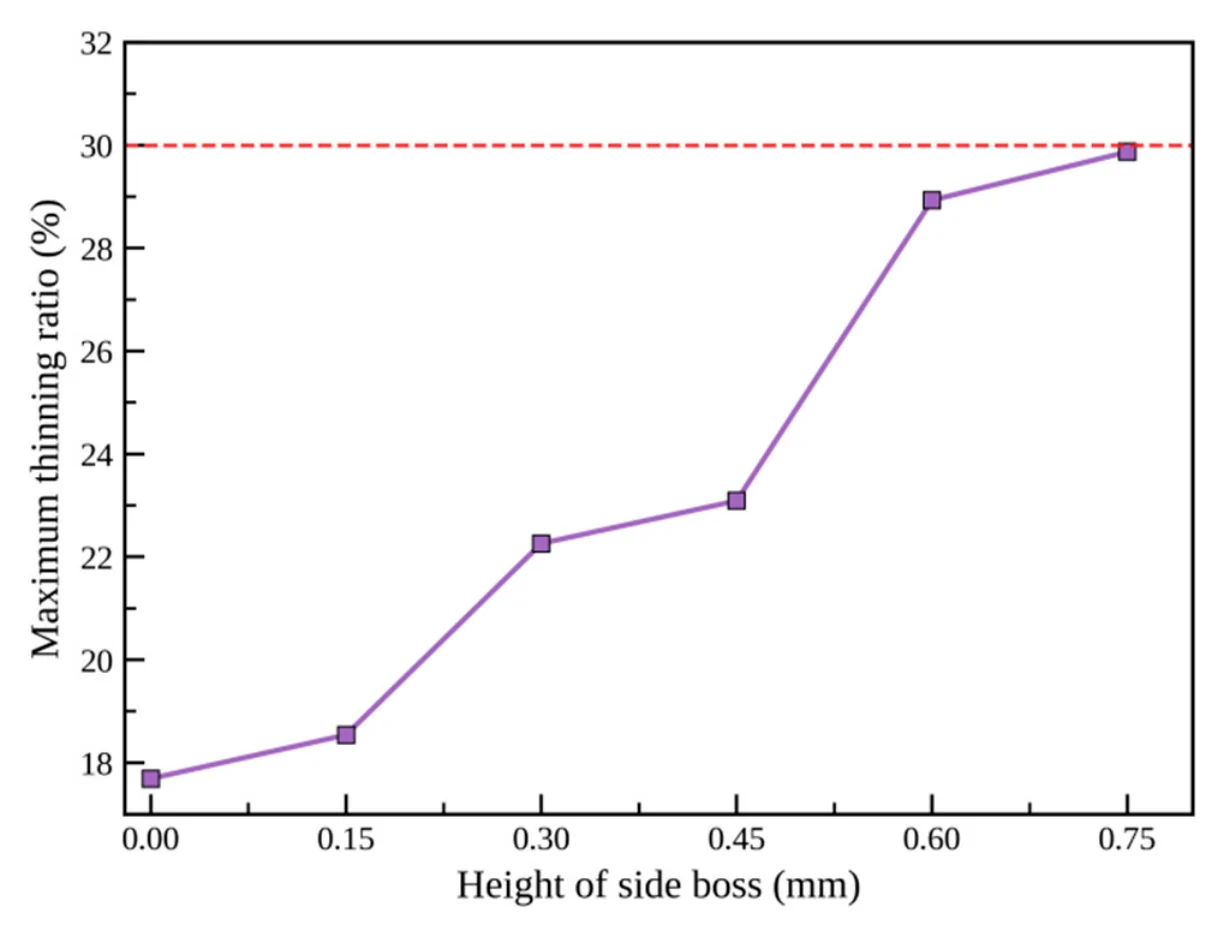
Maximum thinning ratio as a function of side-boss height.

The thickness-field evolution and the associated maximum-thinning trend are compared in Figure 18 and Figure 19. As height increases from 0 to 0.75 mm, the maximum thinning ratio rises from 17.69% to 29.87%. The increase is moderate up to 0.45 mm, where the value is 23.09%, and becomes much steeper between 0.45 and 0.60 mm, reaching 28.93%. At 0.60 mm, isolated high-strain points approach or cross the estimated FLC. At 0.75 mm, the exceedance becomes spatially coherent and is accompanied by 29.87% thinning at the boss top and channel corner, identifying this case as an elevated local-necking-risk configuration.

The trend is physically consistent with increasing local bending–stretching demand and reduced material supply at steeper boss walls. A higher protrusion increases the geometric gradient at the boss root, enlarges the high-curvature contact zone, and increases the material draw-in required to fill the top and sidewall. Once the height exceeds approximately 0.45 mm, the strain field expands from a localized concentration toward a broader high-risk region. The transition between 0.45 and 0.60 mm reflects the combined influence of material response, friction, blank-holder restraint, local radii, and the forming-limit state and provides a practical threshold for process design under the reference conditions.

Industrial assessment additionally considers residual gas tightness, dimensional accuracy, springback, coating integrity, corrosion resistance, and production repeatability. Talebi-Ghadikolaee et al. [13] reported a critical thinning magnitude of 33.45% for an experimentally investigated 0.1 mm stainless-steel stamping condition. The present 29.87% value is only 3.58 percentage points below that reference and is accompanied by a spatially coherent high-risk region.

Accordingly, H_b_ = 0.75 mm is classified as a narrow-margin, high-risk configuration for single-step stamping, while H_b_ = 0.60 mm already marks the onset of rapid forming deterioration. The dashed 30% line in Figure 19 is used as an engineering warning reference that highlights this transition.

Under the present single-step process, side-boss height is the dominant geometric parameter. The H_b_ = 0.15 mm case remains below the estimated FLC with a maximum thinning ratio of 18.54%. The H_b_ = 0.30–0.45 mm cases form a transition range characterized by wrinkling tendency or proximity to the estimated FLC, whereas H_b_ ≥ 0.60 mm requires process modification to restore the forming margin.

### 3.4. Effect of Punch Speed on Formability

Punch speed was varied from 200 to 1700 mm/s while geometry, friction, and blank-holder force were fixed. The speed-dependent response reflects the combined influence of the adopted rate-dependent SS304 flow curves, evolving sheet–tool contact, inertia, and material-flow redistribution.

The maximum thinning ratios at 200, 500, and 800 mm/s are 24.60%, 23.09%, and 23.16%, respectively. The difference between 500 and 800 mm/s is only 0.07 percentage points, confirming a low-sensitivity interval over 200–800 mm/s. The 500 mm/s case gives the lowest discrete thinning value among the sampled speeds.

**Figure 20:**
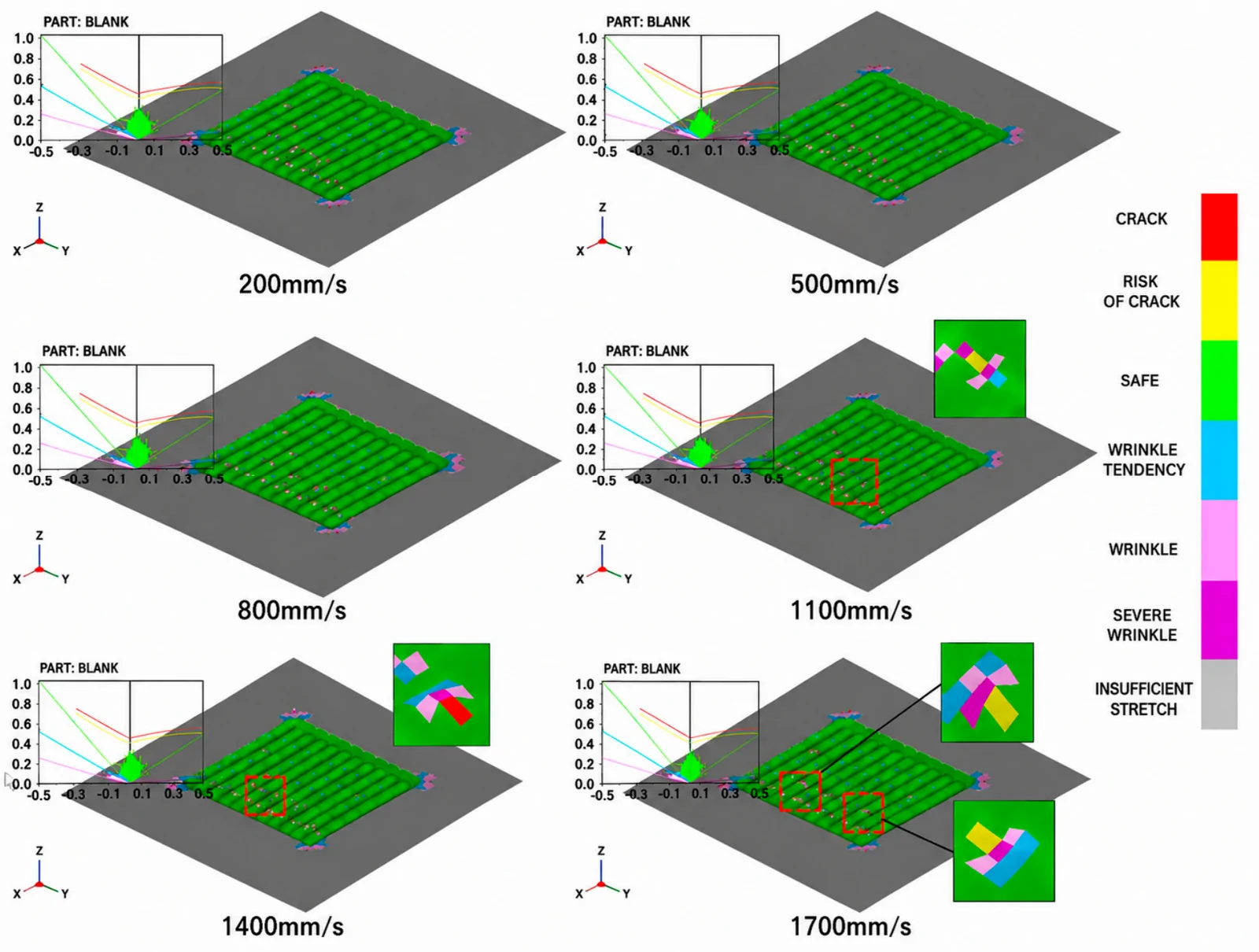
Estimated-FLD maps at different punch speeds.

At 1100 mm/s, the maximum thinning ratio rises to 26.30% and local points near the side-boss roots reach the estimated-FLC warning region. The estimated-FLD maps in Figure 20 show how the elevated-risk region broadens around the root and chamfer at 1400 and 1700 mm/s, while maximum thinning stabilizes at 26.50% and 26.58%, respectively. These results define a transition from a low-sensitivity regime to an elevated local-necking-risk regime near 1100 mm/s.

The transition near 1100 mm/s is consistent with the combined effects of rate-dependent strengthening, accelerated contact evolution, inertial constraint, and reduced redistribution time at the side-boss roots. As the speed increases further, the maximum thinning ratio approaches a plateau near 26.5%, indicating that the deformation pattern becomes increasingly governed by the fixed local geometry and contact path.

**Figure 21:**
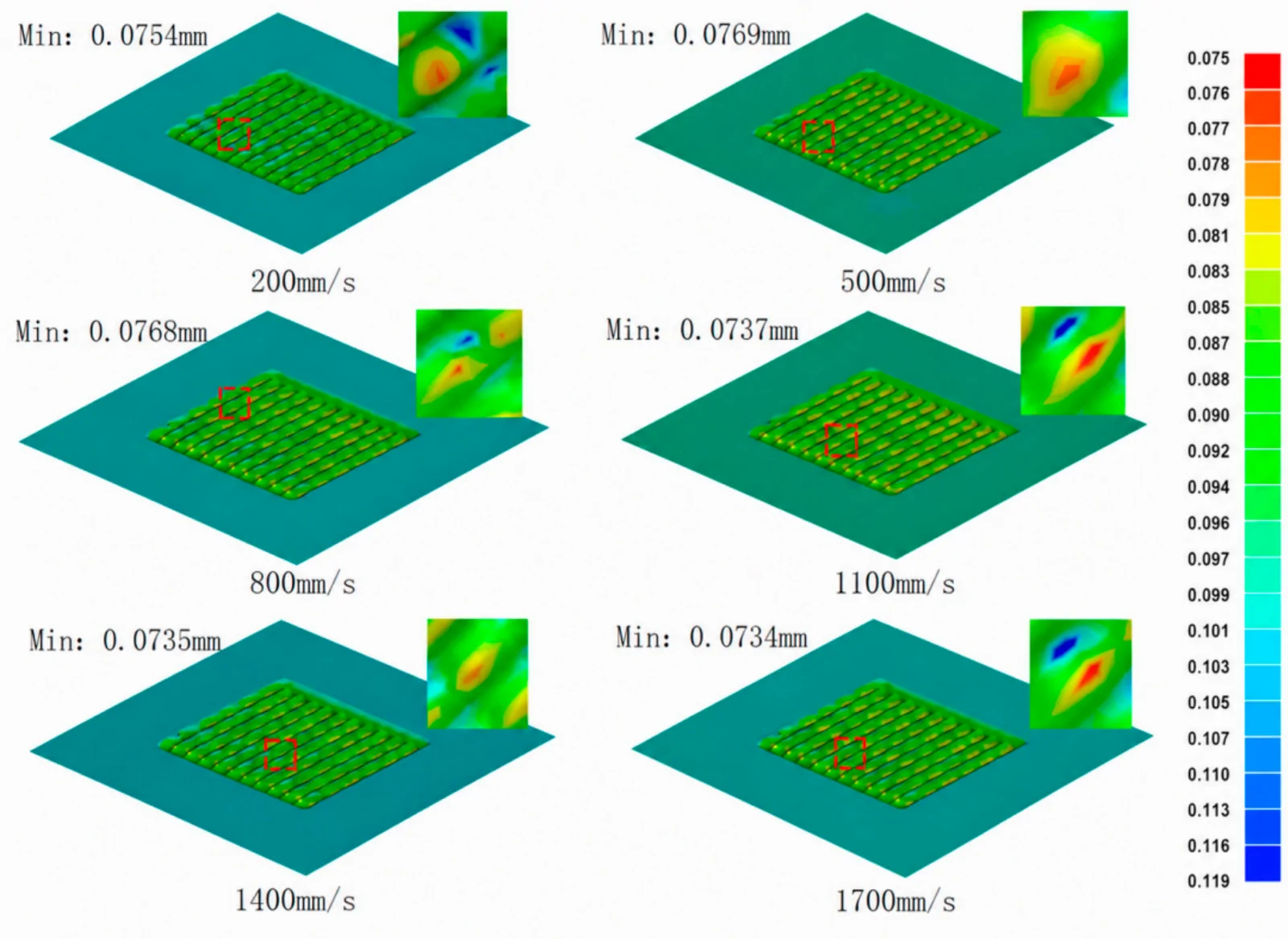
Thickness distributions at different punch speeds.

**Figure 22:**
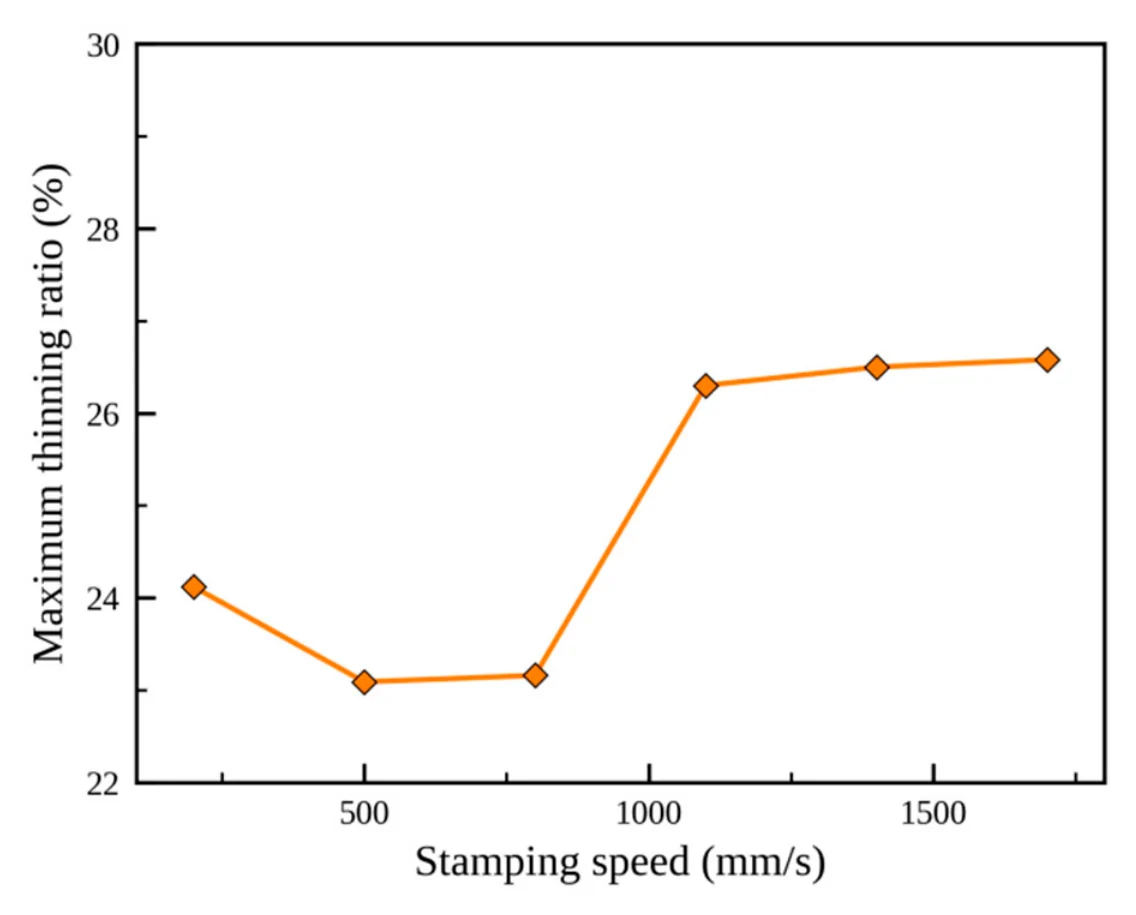
Maximum thinning ratio at different punch speeds.

The corresponding thickness distributions and maximum-thinning trend are compared in Figure 21 and Figure 22. At 200, 500, and 800 mm/s, the maximum thinning ratios are 24.60%, 23.09%, and 23.16%, respectively. Relative to 200 mm/s, the 500 and 800 mm/s cases are lower by approximately 6.14% and 5.85%, while the difference between 500 and 800 mm/s is only 0.07 percentage points (approximately 0.30%). At 1100 mm/s, the ratio increases to 26.30%, which is 13.9% higher than the 500 mm/s case; the values then remain near 26.5% at 1400–1700 mm/s. The quantitative trend identifies 200–800 mm/s as a low-sensitivity interval and ≥1100 mm/s as an elevated-risk interval, with 500 mm/s selected as the representative process baseline.

Accordingly, 200–800 mm/s constitutes a low-sensitivity speed interval under the investigated conditions. The 500 mm/s case is retained as the representative baseline because it gives the lowest sampled thinning value, while 1100 mm/s marks the onset of the elevated-risk regime.

### 3.5. Manufacturing Implications and Design Guidance

The one-factor matrix resolves the dominant first-order effects and their physical coupling. Side-boss height controls the bending–stretching demand and material supply, boss number and pitch govern the overlap of adjacent strain fields, and punch speed modifies rate response, contact evolution, inertia, and redistribution. The factor-specific guidance below therefore applies to μ = 0.125, F_BH_ = 1800 N, the present tooling geometry, and the adopted single-step stamping route.

For the high-severity 0.60–0.75 mm geometries, established forming routes offer different mechanisms for redistributing deformation: multi-stage stamping divides the total shape change into successive increments, hydroforming and flexible-die forming provide distributed support, and warm or hot forming reduces instantaneous flow stress. Their practical selection should balance filling accuracy, springback, surface condition, cycle time, and tooling cost. The quantitative thinning responses, critical locations, and corresponding estimated-FLD interpretations are summarized in Table 6, while the factor-specific design guidance derived from the present simulations is presented in Table 7.

One side-boss set, H_b_ ≤ 0.15 mm, and 200–500 mm/s define the conservative factor-wise forming domain. The performance-oriented M = 5, H_b_ = 0.45 mm configuration combines a 3.02% increase in peak power density and an 11.61% increase in oxygen-distribution uniformity with a maximum thinning ratio of 23.09%, thereby providing a quantitative performance–formability reference. Heights of 0.60 mm or greater and punch speeds of 1100 mm/s or greater form the elevated-risk domain under the investigated conditions.

## 4. Conclusions

This study numerically investigated the single-step stamping formability of 0.1 mm SS304 metallic bipolar plates containing repeated local sidewall bosses within an otherwise straight parallel-channel flow field. The main conclusions are as follows:

(1) The study isolates the forming penalty introduced by repeated sidewall bosses while retaining the global parallel-channel path. The bosses shift the principal high-risk regions from the conventional channel bottom toward boss root fillets, boss tops, local transitions, and channel corners.
(2) Side-boss number produces a non-monotonic response. Maximum thinning is 17.69% for the unmodified baseline and 19.54–27.76% for the modified cases. The two-set case gives the largest scalar peak, whereas the five-set case produces broader estimated high-risk regions; both magnitude and spatial continuity must therefore be considered.
(3) Side-boss height is the dominant investigated geometric factor. Increasing H_b_ from 0 to 0.75 mm raises maximum thinning from 17.69% to 29.87%, a 12.18-percentage-point increase, and leaves a minimum local thickness of approximately 0.0701 mm. Heights of 0.60 mm and above have substantially reduced forming margin under the present single-step process.
(4) Punch speed has a smaller effect over 200–800 mm/s, where maximum thinning varies from 23.09% to 24.60%. The value rises to 26.30% at 1100 mm/s, marking the transition to an elevated-risk regime governed by rate response, contact evolution, inertia, and restricted material redistribution. The 500 mm/s case gives the lowest sampled thinning value and is retained as the representative process baseline. The M = 5, H_b_ = 0.45 mm geometry increases peak power density by 3.02% and oxygen-distribution uniformity by 11.61%, while producing a maximum thinning ratio of 23.09%. This quantified trade-off links flow-field performance directly with stamping formability and provides a practical basis for selecting side-boss geometry and process conditions.

## 5. Limitations and Future Work

The conclusions of this study are based on numerical simulation. Therefore, the predicted thickness distribution and FLD states should be regarded as comparative design indicators. Future work should include physical stamping tests, thickness measurements of formed samples, and comparison between measured and simulated forming results. Such validation is necessary before the proposed design window can be used for actual production tooling.

In addition, other process variables, such as blank-holder force, friction coefficient, die clearance, temperature, and multi-stage forming strategy, should be investigated to improve the robustness of the forming process.

For practical PEMFC applications, the forming feasibility discussed in this paper should eventually be combined with electrochemical performance, corrosion resistance, interfacial contact resistance, and surface coating durability in order to evaluate the overall suitability of side-boss metallic bipolar plates.

## Figures and Tables

**Table 1:** Material parameters adopted for SS304.

Density (g cm^−3^)	Elastic Modulus (MPa)	Poisson Ratio	Yield Strength (MPa)	Hardening Exponent Used for FLC Estimation, *n*
7.85	207,000	0.28	248	0.502

Note: *n* = 0.502 is used as a representative input for the empirical FLC estimation. The finite element post-yield response is defined by the tabulated true stress–plastic strain curves in Figure 5.

**Table 2:** Summary of the numerical model and fixed analysis conditions.

Category	Parameter	Adopted Setting	Role in Analysis
Software and route	CAD / solver	SolidWorks geometry imported into Dynaform/LS-DYNA; 3D double-action forming	Binder Closing followed by punch Drawing
Blank	Dimensions	40 mm × 40 mm × 0.100 mm; active flow-field area 21 mm × 20 mm	Provides material for draw-in around the channel region
Reference channel	Geometry	S = W = 1.0 mm; channel depth = 0.50 mm; R = r = 0.10 mm; draft angle = 5°	Held constant to isolate side-boss effects
Variables	Parameter matrix	M = 0–5; H_b_ = 0–0.75 mm; punch speed = 200–1700 mm/s	Resolves first-order geometry and speed trends
Blank elements	Formulation	Belytschko–Tsay shell; five through-thickness integration points	Captures coupled membrane and bending deformation
Tooling	Treatment	Rigid punch, binder, and die	Defines a stable reference tool geometry
Mesh	Local refinement	0.025 mm in boss roots, fillets, tops, and corners; up to 0.40 mm in remote regions	Resolves high-curvature strain gradients efficiently
Material	Elastic properties	ρ = 7.85 g cm^−3^; E = 207 GPa; ν = 0.28; σ_y_ = 248 MPa	Provides the reference elastic response
Plasticity	Yield / hardening	Isotropic J_2_/von Mises yield; tabulated true stress–plastic strain curves	Defines macroscopic plastic flow and strain localization
Rate input	Available curves	Nominal tensile strain rates 0.0005–0.1 s^−1^ [45]	Represents the rate-dependent material response used in the punch speed comparison.
Anisotropy	FLC input	r = 1.0 as a consistent isotropic reference in the FLC estimator	Provides a common directional basis across all cases.
Forming assessment	Local-necking indicator	Major–minor strain states evaluated relative to the estimated FLC	Identifies local-necking tendency and high-risk regions
Contact	Formulation	Penalty contact; penalty factor 0.15; Coulomb friction μ = 0.125	Defines common sheet–tool interaction
Blank restraint	Blank-holder force	q = 3 MPa; projected area ≈ 600 mm^2^; F_BH_ = 1800 N	Controls material draw-in under the reference condition
Tool positioning	Closing gap	0.11 mm	Sets the initial tool approach and closure
Forming limit	Estimated FLC	Dynaform empirical estimator using thickness, *n* = 0.502, and r = 1.0	Provides a consistent strain-limit reference
Process temperature	Thermal condition	Constant-temperature cold-stamping condition	Maintains a common thermal reference for all punch speed cases.
Outputs	Assessment	Thickness distribution, maximum thinning ratio, and estimated-FLD state	Quantifies thinning and critical-location evolution

Note: Except for side-boss number, side-boss height, and punch speed, all geometric, material, contact, restraint, and solver settings were maintained unchanged. This consistent baseline isolates the first-order response of each investigated parameter.

**Table 3:** Operational definitions used for the estimated-FLD states.

Reported State	Operational Definition in This Study	Engineering Interpretation
Below the estimated FLC	Evaluated tensile strain points remain below the estimated curve without visible local crossing	Tensile strain states remain within the estimated forming margin
Wrinkling tendency	Compressive or instability-related regions are indicated in the Dynaform FLD map	Compressive instability is locally activated
Near the estimated FLC	Local strain points approach the curve or warning region without clear repeated crossing	The local forming margin is reduced
Estimated local-necking tendency	One or a limited number of local points reach or cross the estimated FLC	Localized necking is likely to initiate at the identified region
Elevated estimated local-necking risk	Repeated or spatially clustered points reach/cross the curve in a consistent high-curvature region, generally with substantial thinning	Spatially coherent local-necking risk is accompanied by substantial thinning

**Table 4:** Quantitative mesh-sensitivity results.

Local Minimum Element Size (mm)	Maximum Thinning Ratio (%)	Difference from 0.025 mm	Assessment
0.100	43.18	+4.81%	1 element across a 0.1 mm fillet; local gradients under-resolved
0.075	41.49	+0.70%	1.3 elements across a 0.1 mm fillet; response near the convergence plateau
0.050	41.64	+1.07%	2 elements across a 0.1 mm fillet; suitable for preliminary screening
0.025	41.20	Reference	4 elements across a 0.1 mm fillet; selected as the conservative final mesh

**Table 5:** Comparison of the present results with published experimental–numerical stamping studies.

Material and Method	Published Experimental–Numerical Evidence	Consistency with Present Results	Benchmark Study
SS304 metallic bipolar plate; Dynaform FE and stamping experiment	Numerical and experimental results reproduced the principal thickness/defect trends under changing process conditions	Consistent with the Dynaform-based thickness and speed-response trends	Hu et al. [30]
Stainless-steel bipolar plate; FE plus experiment	Approximate thinning-related critical level of 33.45%; critical elements associated with plane-strain tension	Provides a quantitative reference for the present 29.87% upper-bound result	Talebi-Ghadikolaee et al. [13]
Approximately 0.1 mm 316L sheet; micro-stamping experiment, FE, and experimental FLD	Severe thinning and failure initiation concentrated in high-curvature sheet–tool contact regions; local necking-governed failure	Consistent with boss root, boss top, transition, and corner localization	Zhang et al. [17]
0.1 mm SS304 side-boss parallel channel; Dynaform	17.69–29.87% thinning; critical region migrates to boss root/top/corner; 200–800 mm/s comparable	Defines the new local side-boss geometry and its quantified forming trends	Present study
SS316L and CP-Ti; physical stamping and FE analysis	Blank-holder restraint reduced wrinkling; severe thinning occurred at sheet–tool contact transitions; maximum springback deviation 25.12%	Consistent with the coupled effects of restraint, contact transition, speed, and recovery	Acar et al. [18]

**Table 6:** Quantitative comparison of thinning response and estimated-FLD states under the investigated conditions.

Variable	Value	Maximum Thinning Ratio (%)	Location of Minimum Thickness	Estimated-FLD Interpretation
Number of side-boss sets	0	17.69	Channel-bottom centre	Below the estimated FLC
	1	19.54	Side-boss root chamfer	Below the estimated FLC
	2	27.76	Side-boss root chamfer	Estimated local-necking tendency
	3	26.00	Side-boss root and top	Near the estimated FLC
	4	22.82	Side-boss top	Near the estimated FLC
	5	23.09	Side-boss top and flow-field edge	Elevated estimated local-necking risk
Side-boss height (mm)	0	17.69	Channel-bottom centre	Below the estimated FLC
	0.15	18.54	Side-boss root chamfer	Below the estimated FLC
	0.30	22.90	Side-boss root chamfer	Wrinkling tendency
	0.45	23.09	Side-boss top	Near the estimated FLC
	0.60	28.93	Side-boss top	Estimated local-necking tendency
	0.75	29.87	Side-boss top and channel corner	Elevated estimated local-necking risk
Punch speed (mm/s)	200	24.60	Channel–sidewall transition	Below the estimated FLC
	500	23.09	Side-boss top	Below the estimated FLC
	800	23.16	Side-boss top	Near the estimated FLC
	1100	26.30	Side-boss root	Estimated local-necking tendency
	1400	26.50	Side-boss root and chamfer	Elevated estimated local-necking risk
	1700	26.58	Side-boss root	Elevated estimated local-necking risk

Note: The estimated-FLD states follow the definitions in Table 3 and are evaluated together with thinning magnitude and critical-region location to classify forming risk.

**Table 7:** Factor-specific design guidance derived from the present simulations.

Parameter	Lower Risk	Transition	High Risk
Number of side-boss sets	M = 1	M = 3–4	M = 2 and M = 5
Side-boss height	H_b_ ≤ 0.15 mm	H_b_ = 0.30–0.45 mm	H_b_ ≥ 0.60 mm
Punch speed	200–500 mm/s	800 mm/s	≥1100 mm/s
Performance-motivated geometry	—	M = 5, H_b_ = 0.45 mm	—
Overall	M = 1; H_b_ ≤ 0.15 mm; 200–500 mm/s	M = 5; H_b_ = 0.45 mm	H_b_ ≥ 0.60 mm; speed ≥ 1100 mm/s

Note: The guidance is based on μ = 0.125, F_BH_ = 1800 N, the present tooling geometry, and the adopted single-step stamping conditions.

## Data Availability

The original contributions presented in this study are included in the article. Further inquiries can be directed to the corresponding authors.
